# Characterization of the Differential Tolerance of Two *Triticum durum* Cultivars to Short-Term Cadmium-Induced Stress

**DOI:** 10.3390/plants15030418

**Published:** 2026-01-29

**Authors:** Mohamed-Amine Hamzaoui, Ángel Barón-Sola, Michiel Huybrechts, Mohamed Banni, Ann Cuypers, Luis E. Hernández, Cristina Ortega-Villasante

**Affiliations:** 1 Laboratory of Plant Physiology, Department of Biology, Universidad Autónoma Madrid, Darwin 2, ES28049 Madrid, Spain; aminehamzaoui23@gmail.com (M.-A.H.); angbaron@ucm.es (Á.B.-S.); cristina.ortega@uam.es (C.O.-V.); 2 Centre for Environmental Sciences, Universiteit Hasselt, BE-3590 Diepenbeek, Belgium; michiel.huybrechts@uhasselt.be (M.H.); ann.cuypers@uhasselt.be (A.C.); 3 Higher Institute of Biotechnology, University of Monastir, Monastir BP 74 5000, Tunisia; m_banni@yahoo.fr

**Keywords:** durum wheat cultivars, cadmium toxicity, oxidative stress, seedling screening

## Abstract

Cadmium (Cd) is a toxic heavy metal that impairs plant growth and induces oxidative stress. In this study, we compared the physiological, biochemical, and molecular responses of two durum wheat (*Triticum turgidum* ssp. *durum*) cultivars, Razek and Chili, to Cd stress. Seedlings were exposed to 0, 5, and 50 µM Cd (Cd^2+^; supplied as CdCl_2_) under controlled hydroponic and Petri assay conditions. Cd reduced radicle elongation, biomass accumulation, and water uptake in both cultivars, but the relative inhibition of growth was lower in Razek than in Chili, indicating a better capacity to maintain growth under Cd stress. This was accompanied by milder oxidative stress symptoms and more stable antioxidant enzyme activity, particularly for catalase (CAT) and ascorbate peroxidase (APX). Gene expression analyses revealed that Razek maintained a higher expression of antioxidant and stress-related genes under acute Cd stress, while Chili exhibited pronounced downregulation. Histochemical analyses showed increased H_2_O_2_ accumulation and lignin deposition in Chili roots, suggesting a stronger stress response. Notably, Chili also showed a sharp depletion of reduced glutathione (GSH) under high Cd concentrations, with limited upregulation of GSH synthesis and phytochelatin-related genes. Together, these findings indicate that Razek activates more efficient detoxification, redox regulation, and hormonal signaling pathways under Cd stress, indicating its potential suitability for cultivation in slightly Cd-contaminated soils.

## 1. Introduction

Durum wheat (*Triticum turgidum* ssp. *durum*) is the main staple cereal crop in Tunisia, occupying over 50% of the country’s arable land. To maintain high yields, this crop requires intensive phosphate fertilization—which is readily available as Tunisia is one of the world’s major phosphate producers—but fertilizers often contain elevated cadmium (Cd) [1]. In well-aerated environments and under our experimental conditions, Cd is mainly present as the divalent cation Cd^2+^; for readability, we refer to this species as “Cd” hereafter. Reported concentrations can reach 53 mg·kg^−1^, above the global average of 18 mg·kg^−1^ and far exceeding the EU soil protection threshold of 1.5 mg·kg^−1^ [2,3]. Continuous use of Cd-containing fertilizers has contributed to the accumulation of this toxic metal in agricultural soils, posing increasing risks to crop production and food safety. Cd is a non-essential and highly toxic element, classified as a Group 1 carcinogen. Its accumulation in soils impairs plant development and crop production, and may affect human health through the food chain [4].

In plants, Cd uptake occurs unintentionally via essential metal transporters, such as members of the ZIP and Nramp families, due to its chemical similarity to zinc (Zn), iron (Fe), and manganese (Mn) [5]. The accumulation of Cd in plant cells leads to toxic effects, such as limited germination, inhibition of biomass, and impairment of plant growth and development. These effects are associated with the limitation of essential metabolic and physiological processes, including respiration and photosynthesis [6]. Some of these symptoms may be associated with the overproduction of reactive oxygen species (ROS)—particularly superoxide (O_2_^●−^), hydrogen peroxide (H_2_O_2_), and hydroxyl radicals (^●^OH)—via various mechanisms, including altered antioxidant enzymatic activities and antioxidant metabolites concentrations [7], which results in cellular damage such as lipid peroxidation, protein denaturation, and alteration of membrane integrity. A putative source of ROS in plant cells is the induction of the plasma-membrane-associated NADPH oxidase family (RBOH; NOX), which initially releases O_2_^●−^ in the apoplast that in turn can trigger stress signaling [8].

In response to the accumulation of ROS, plants activate antioxidant defense mechanisms to restore the cellular redox balance [9]. The ascorbate–glutathione cycle enables the scavenging of ROS. This process involves the conversion of O_2_^●−^ to H_2_O_2_ by superoxide dismutase (SOD), followed by the reduction of H_2_O_2_ to H_2_O by ascorbate peroxidase (APX) using ascorbate (ASA), which is fully oxidized to dehydroascorbate (DHA) in the final steps of this process. DHA is reduced back to ASA by DHAR, resulting in the consumption of glutathione (GSH) and the production of oxidized glutathione (GSSG). The cellular GSH pool is then maintained by glutathione reductase (GR), which reduces oxidized glutathione (GSSG) back to GSH using NADPH as an electron donor [10]. In addition, catalase (CAT) can also scavenge H_2_O_2_ via its dismutation to H_2_O and O_2_. This enzyme is mostly distributed in peroxisomes, but some isoforms are present in roots associated with lignification of vascular tissues [11].

Lignin deposition results from the polymerization and cross-linking of monolignols, a process influenced by ROS accumulated in the apoplast. This leads to cell wall stiffening, a response that is enhanced under Cd stress [12,13]. In addition to cell wall stiffening, lignin contains various functional groups that can bind Cd, resulting in a decrease in Cd mobility within root plant cells [14]. Recent studies have shown the interplay between ROS production, stress-related phytohormones, and lignin biosynthesis in drought-stressed wheat plants, where cell wall reinforcement may be required to counteract damage in cells suffering homeostasis imbalances under abiotic stress [15].

In addition to the relevant part of GSH in counteracting metal toxicity as an antioxidant, GSH plays an important role in Cd tolerance as a precursor of phytochelatins (PCs) [16,17], a group of oligopeptide biothiols with the general γ(Glu-Cys)_n_-Gly (*n* = 2–5) structure that are synthesized by phytochelatin synthases (PCSs) [18]. Thus, PCs are a diverse family of Cys-rich peptides that form complexes with Cd, which are subsequently sequestered in the vacuole to reduce their toxicity within the plant cell [19]. Another mechanism to counteract Cd toxicity is related to the expression of several small heat shock proteins (sHSPs), which are known to protect essential proteins from Cd-induced denaturation [20]. These small (12–40 kDa) proteins play a crucial role by assisting in the proper folding of denatured proteins caused by oxidative damage under different environmental stresses [21]. They were overexpressed in response to Cd in rice [22], poplar [23], *Vigna* [24], or *Arabidopsis* [25]. To replace cellular components damaged by oxidative stress, autophagy is considered an essential mechanism that also plays a crucial role in mitigating the deleterious effects of toxic metals such as Cd by recycling non-functional cellular material and contributing to the maintenance of cellular homeostasis [26]. Several autophagy genes (ATGs) are required for this tightly regulated process [27], among which the ubiquitin-like protein ATG8 plays a central role in autophagy induction [28]. The expression of ATG8 is readily upregulated in response to various biotic and abiotic stresses [29].

The aim of this work was to compare two durum wheat cultivars (Razek and Chili), frequently grown in Tunisia, and to provide the first evidence of cultivar-dependent differences in their responses to Cd stress. We hypothesized that these differences are associated with distinct oxidative stress responses and antioxidant defense regulation, which can be reliably detected at the seedling stage under controlled exposure conditions. Therefore, we used a hydroponic system with defined Cd concentrations to analyze both cultivars’ responses, focusing on oxidative stress-related parameters, including the expression of antioxidant genes. This controlled screening represents a first step toward selecting cultivars with improved Cd tolerance for subsequent validation under more applied (soil and field) conditions, ultimately contributing to minimizing Cd-related yield losses in Cd-affected agricultural areas.

## 2. Results

### 2.1. Effect of Cd on Plant Growth and Biomass

**Figure 1:**
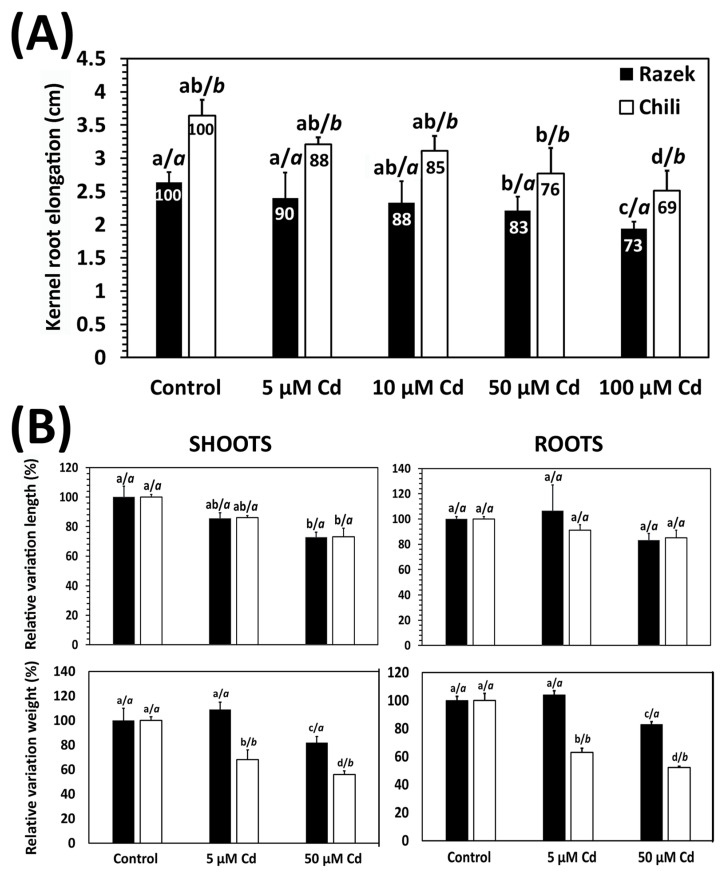
Effects of Cd on Razek and Chili durum wheat biomass: (**A**) Germination test measuring radicle elongation (cm) of kernels (seedlings) after 24 h in control (0), 5, 10, 50, and 100 µM Cd solutions. Values inside the bars indicate relative growth variation (%). (**B**) Relative variation (%) in shoot and root length (upper panel) and fresh weight (lower panel) after 72 h treatment in the hydroponic experiment. Mean ± standard deviation (S.D.) of five biological replicates; letters on top of the bars indicate significant differences between treatments (left), and between cultivars (right) (*p* < 0.05).

A rapid kernel (seed) germination test revealed contrasting responses to Cd between the two durum wheat cultivars, Chili and Razek. In absolute terms, Chili exhibited significantly greater radicle elongation under Cd exposure than Razek (Figure 1A; Supplementary Figure S1). However, when radicle elongation was expressed relative to the corresponding control (i.e., Cd-induced inhibition), Razek was slightly less affected by Cd than Chili, suggesting a lower Cd sensitivity of this cultivar (Figure 1A). To further investigate these cultivar-dependent responses, 15-day-old seedlings of both cultivars were grown hydroponically and exposed to Cd (0, 5, and 50 µM) for 72 h to analyze Cd-sensitive physiological parameters (Supplementary Figure S2). Under control conditions, Chili generally displayed higher absolute values (cm or g per plant) than Razek (Supplementary Table S3). When considering relative variations in organ length, no significant differences between cultivars were observed under Cd stress (Figure 1B, upper panel). In contrast, clearer cultivar-specific differences emerged for biomass-related traits: the relative reduction in fresh organ weight with increasing Cd doses was significantly greater in Chili than in Razek (Figure 1B; lower panel).

### 2.2. Physiological Responses to Cd

**Figure 2:**
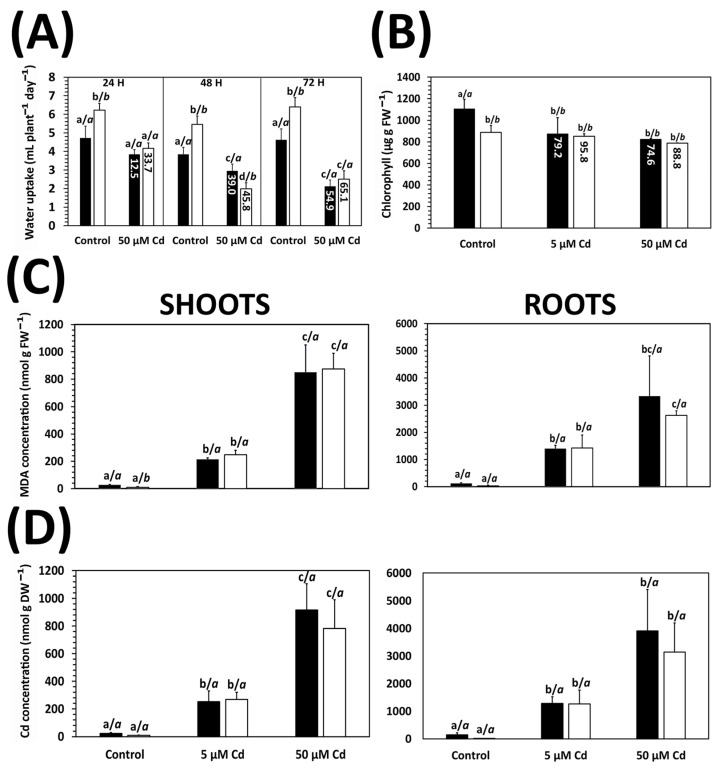
Effects of Cd (control (0), 5, and 50 µM) on Razek (black) and Chili (white) durum wheat: (**A**) Water uptake (mL H_2_O plant^−1^ day^−1^) after 24, 48, and 72 h. (**B**) Chlorophyll concentration (µg/gFW). (**C**) Lipid peroxidation as accumulation of the by-product MDA (nmol/gFW). (**D**) Cd concentration (µg/gDW) in shoots and roots after 72 h treatment. Mean ± S.D. of three biological replicates. Values inside the bars indicate relative variation (%) compared with controls. Letters indicate significant differences between treatments (left), and between the cultivars (italic) (*p* < 0.05).

The reduction in radicle elongation and fresh weight observed in Cd-treated plants could be related to a limitation of H_2_O availability, which primarily affects cell elongation driven by H_2_O turgor pressure, as occurs in wheat plants exposed to drought [30]. Exposure to Cd resulted in a significant decrease in water uptake from the nutrient solution, a negative effect that was already observed after 24 h (Figure 2A). Regarding the differences between cultivars, Chili usually showed higher water uptake rates than Razek, even in the presence of Cd, corresponding to the higher biomass of this cultivar. H_2_O uptake decreased with exposure time, with the most pronounced reduction observed after 72 h with 50 μM Cd (Figure 2A). Notably, relative H_2_O uptake was more strongly inhibited in Chili than in Razek at all time points (refer to values within bars in Figure 2A).

Cd exposure led to a dose-dependent reduction in chlorophyll concentration, with no significant differences between both cultivars (Figure 2B). This was accompanied by the onset of oxidative stress, as evidenced by the increase in lipid peroxidation with Cd dose in both the shoots and roots (Figure 2C). However, there were no significant differences between Razek and Chili. The appearance of oxidative stress symptoms corresponded to the accumulation of Cd in the plants, which increased in a Cd dose-dependent manner (Figure 2D). The root is the first organ to receive Cd from the nutrient solution, resulting in the expected 4–6 times higher accumulation of Cd in roots compared to shoots (Figure 2D). Finally, there were no apparent varietal differences, as both cultivars accumulated similar levels of Cd in the roots and shoots in all treatments.

Biothiols are an important component of Cd tolerance in plants, both as antioxidants and as metal chelating agents. There were no significant differences in the concentration of cysteine (Cys) between metal treatments and cultivars (Table 1). In contrast, glutathione (GSH) was the most abundant biothiol in shoots and roots, and its content increased with Cd concentration in Razek, where a strong induction was observed at 50 µM Cd. Chili showed a moderate increase at 5 µM Cd but a marked decline at 50 µM Cd in roots, with values below 30% of those found in Razek, suggesting a differential redox regulation between cultivars (Table 1). The depletion of GSH in Chili roots under Cd stress was further confirmed in a complementary time-course experiment, in which root GSH concentration declined from approximately 80 nmol·g^−1^ FW to nearly half that value after 96 h of exposure to 50 µM Cd (Supplementary Table S4). Phytochelatins (PCs) were only detected in plants exposed to Cd, with the highest concentrations found in roots. Both PC2 and PC3 levels increased in a Cd dose-dependent manner (Table 1). No significant differences were found in the PC2 concentrations between shoots and roots. However, PC3 levels in roots were significantly higher in Razek than in Chili under 5 µM Cd treatment (Table 1). In the time-course experiment, exposure of Chili plants to 50 µM Cd also induced accumulation of PC2 and PC3, with both reaching maximum levels after 96 h of treatment (Supplementary Table S4).

### 2.3. Redox Enzymes and Stress-Related Proteins

**Figure 3:**
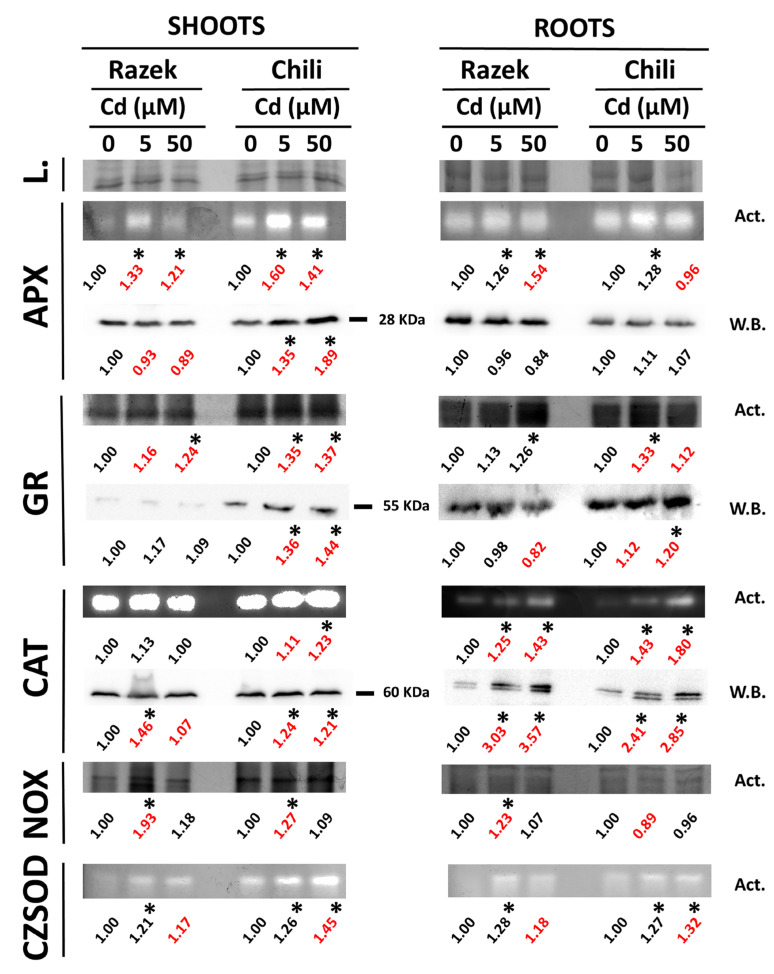
Enzymatic activities (Act.) and Western blot (W.B.) of redox enzymes: APX (ascorbate peroxidase), GR (glutathione reductase), CAT (catalase), NOX (NADPH-oxidase), and CZSOD (Cu/Zn-superoxide dismutase) in roots and shoots of Razek and Chili durum wheat exposed to Cd (0, 5, and 50 μM) for 72 h. Numbers shown below each lane represent relative band intensity estimated by densitometry, normalized to the corresponding control (0 µM Cd) set to 1.00. These values are provided for semi-quantitative comparison only. Asterisks highlight ±20% variation with the control, and red numbers indicate relevant differences between cultivars.

In response to Cd-induced oxidative stress and associated biothiol changes, we examined the involvement of key redox enzymes in the shoots and roots of both cultivars. A major APX isoform band was detected, likely corresponding to cytosolic APX, as supported by a similar signal pattern observed with the α-cAPX antibody in the immunoblot (Figure 3). Shoot APX activity increased in both Razek and Chili under 5 µM Cd, followed by a slight decline at 50 µM Cd, although only Chili showed cAPX accumulation in shoots under Cd stress. In roots, APX activity increased in a Cd dose-dependent manner in Razek, while Chili showed a slight increase at 5 µM Cd, followed by a decrease at 50 µM Cd. However, these changes were not accompanied by notable differences in cAPX protein levels in either cultivar (Figure 3).

GR contributes to maintaining the GSH balance required to counteract ROS, and its activity generally mirrored that of APX. In shoots, GR activity increased in Cd-treated plants, particularly in Chili (Figure 3). In roots, the highest GR activity was observed under 50 µM Cd in Razek, whereas in Chili, it peaked at 5 µM Cd and declined at the highest dose. GR protein levels (α-GR immunodetection) remained relatively unchanged in Razek, while they increased in both the shoots and roots of Chili in response to Cd (Figure 3). CAT activity followed a similar pattern, with a marked dose-dependent increase in the roots of both cultivars, particularly in Chili, partially reflecting elevated CAT protein levels. In contrast, NADPH oxidase activity increased only at the moderate Cd dose (5 µM), notably in Chili shoots and Razek roots, suggesting transient ROS production under moderate stress. Finally, the major SOD activity band, identified as the Cu/ZnSOD isoform by the KCN-H_2_O_2_ inhibitor [31], showed a cultivar-specific response: in Chili, its activity increased progressively with Cd concentration in both the shoots and roots, whereas in Razek, the response peaked at 5 µM Cd and did not increase further at higher doses (Figure 3). The responses of CAT and Cu/ZnSOD to Cd in Chili were confirmed in the supplementary time-course experiment (Supplementary Figure S3A). CAT showed a marked increase in both activity and protein levels, as detected by α-CAT immunoblotting, while Cu/ZnSOD activity displayed only a modest induction at the longest exposure time (96 h).

**Figure 4:**
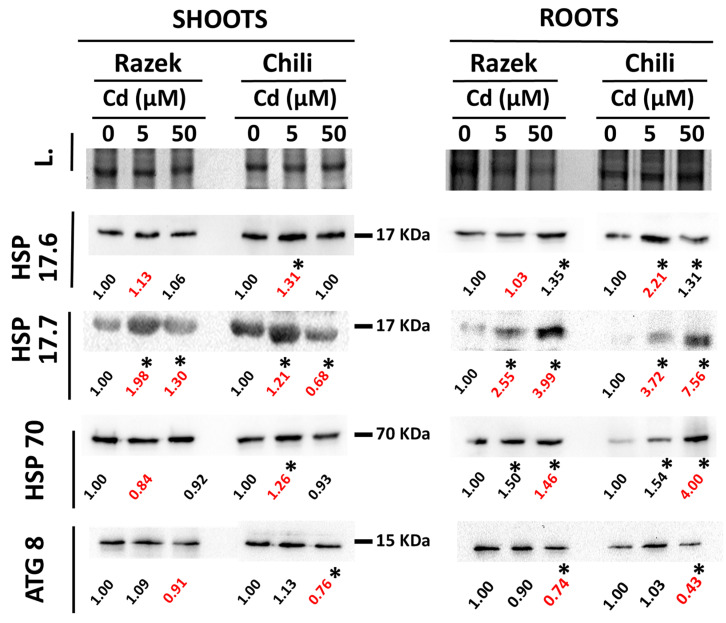
Western blot of HSP 17.6 kDa, 17.7 kDa, and 70 kDa, and ATG 8 in shoots and roots of Razek and Chili durum wheat exposed to Cd (0, 5, and 50 µM) for 72 h. The numbers indicate the relative change in intensity of the bands, the asterisks highlight ±20% variation with the control, and the red numbers indicate relevant differences between cultivars.

Stress-related proteins were analyzed by immunodetection using antibodies against HSP17.6, HSP17.7, and HSP70. Overall, Cd exposure increased the abundance of these proteins, with differences depending on cultivar, organ, and Cd concentration (Figure 4). In shoots, HSP17.7 levels rose with increasing Cd in Razek but declined in Chili at 50 µM Cd. In contrast, HSP17.6 and HSP70 increased only at 5 µM Cd in Chili shoots. In roots, HSP17.7 showed dose-dependent induction in both cultivars, with a stronger response in Chili, suggesting greater protein stress. HSP17.6 and HSP70 followed a similar pattern in Chili roots, showing stronger induction with increasing Cd than in Razek (Figure 4). Similar responses occurred in the time-course experiment, where Chili plants accumulated HSP17.7 and HSP70 in both the shoots and roots after 96 h of exposure to 50 µM Cd (Supplementary Figure S3A). Finally, the autophagy-related ATG8 protein showed minimal changes in response to Cd, although a slight reduction was observed in the roots of both cultivars at 50 µM Cd, particularly in Chili (Figure 4).

### 2.4. Cellular Analyses of Redox Imbalance and Lignin Deposition

**Figure 5:**
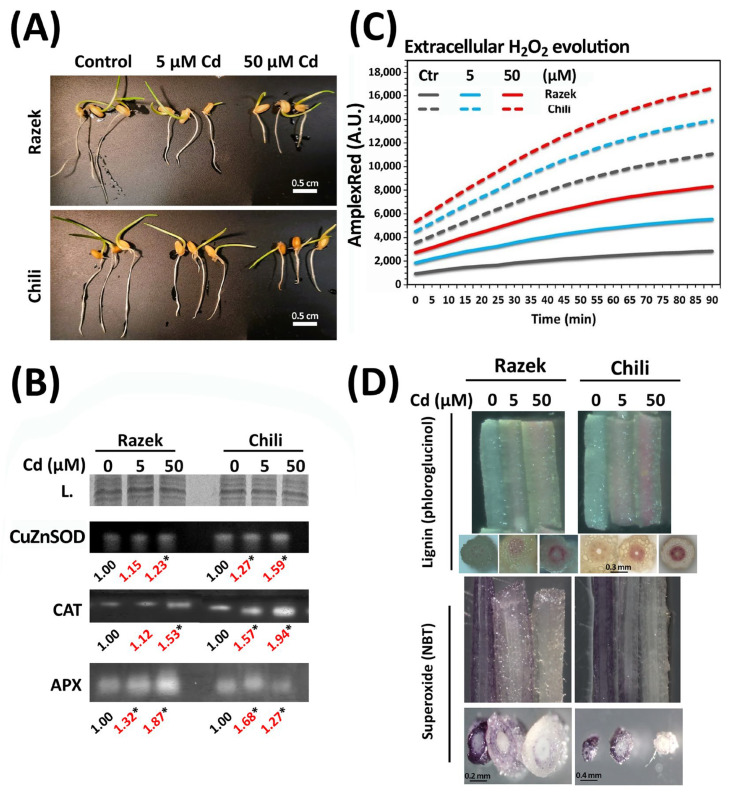
(**A**) Growth of biomass and roots after 3 days of germination of two durum wheat cultivars, Razek and Chili, under control conditions, 5 µM, and 50 µM of Cd. (**B**) Enzymatic activity of CuZnSOD (Cu/Zn-superoxide dismutase), CAT (catalase), and APX (ascorbate peroxidase) in roots of Razek and Chili seedlings exposed to Cd (0, 5, and 50 µM for 24 h). The numbers indicate the relative change in intensity of the bands, the asterisks highlight ±20% variation with the control, and the red numbers indicate relevant differences between cultivars. (**C**) Extracellular H_2_O_2_ evolution (arbitrary fluorescence units (A.U.) of AmplexRed) with time (min) in Razek and Chili. (**D**) Sections (longitudinal and transversal) of wheat roots for detection of superoxide and lignin.

To further investigate the contrasting antioxidant responses of the two durum wheat cultivars under Cd stress, we characterized tissue-level responses in seedlings exposed to 0, 5, or 50 µM Cd for 24 h after two days of germination under control conditions. As shown in Figure 5A, root length decreased in both cultivars in a dose-dependent manner, with severe inhibition at 50 µM Cd. Although Razek exhibited shorter roots overall, it was less affected by Cd than Chili, consistent with the observations in Figure 1A. Enzymatic assays revealed stronger induction of Cu/ZnSOD and CAT activities in Chili than in Razek (approximately 30% higher), while APX activity in Chili peaked at 5 µM Cd and slightly declined at 50 µM (Figure 5B). Varietal differences were also evident in extracellular H_2_O_2_ production, which increased with Cd dose and was consistently higher in Chili root sections (Figure 5C). Reduced NBT staining in response to Cd treatment in both cultivars indicates decreased superoxide (O_2_^•−^) levels, likely due to enhanced SOD activity catalyzing its conversion to H_2_O_2_ (Figure 5D). Concurrently, lignin deposition—as detected by phloroglucinol—increased in response to Cd, with greater accumulation in Chili, possibly due to elevated H_2_O_2_ levels driving monolignol cross-linking. This inverse relationship between O_2_^•−^ levels and lignin accumulation in Chili roots was confirmed in the time-course experiment, where extended exposure to 50 µM Cd (96 h) led to further reductions in NBT staining and increased phloroglucinol signals (Supplementary Figure S3B).

### 2.5. Stress-Related Gene Expression

**Figure 6:**
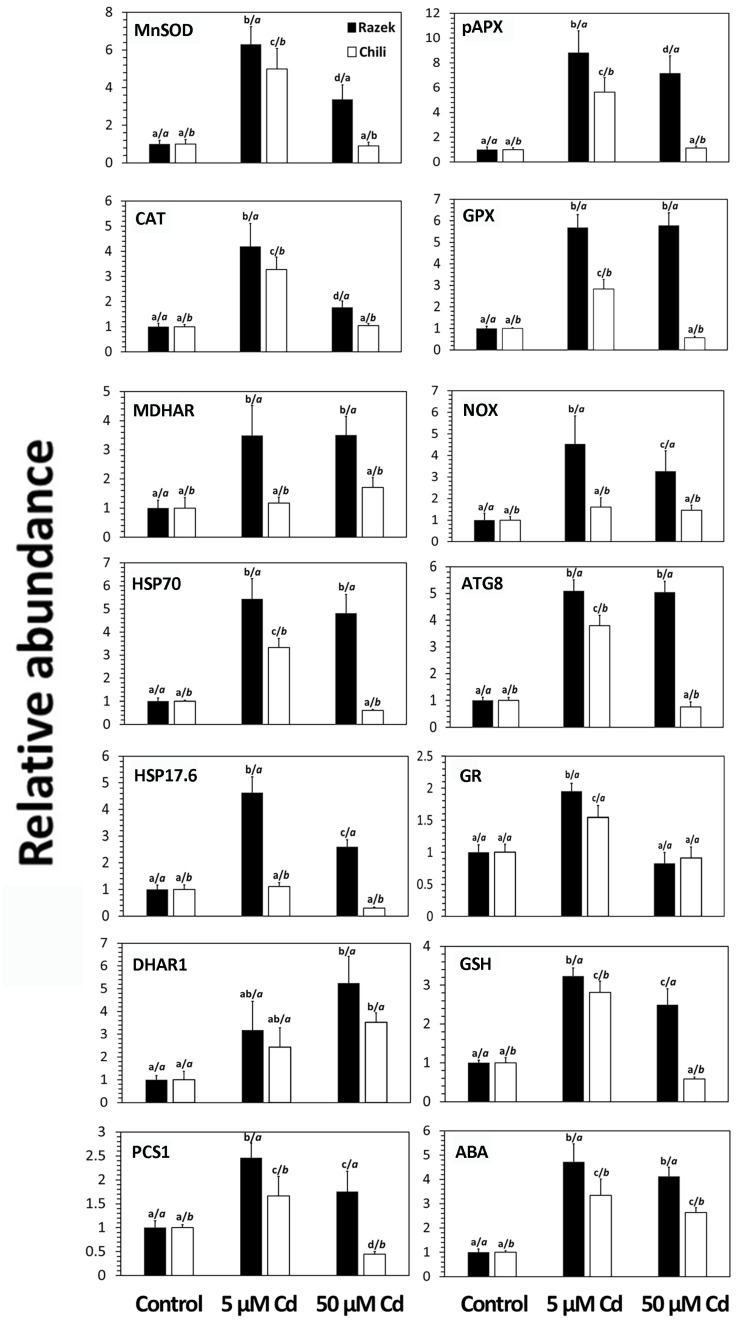
Expression levels of antioxidative and stress-related genes in leaves of two wheat cultivars grown under Cd-exposed conditions (0, 5, and 50 µM for 72 h). See Supplementary Materials (Tables S2 and S5) for further information and details. Values represent the mean ± S.D. of five biological replicates relative to the control condition (set at 1.00); letters on the left indicate significant.

To complete the characterization of oxidative balance in Razek and Chili under Cd stress, we analyzed the expression of genes involved in oxidative stress responses and regulation, revealing clear differences between the two cultivars (Figure 6, Supplementary Table S5). Antioxidant-related genes such as *MnSOD*, *APX*, *CAT*, *GPX*, and *MDHAR* were upregulated under Cd stress, with peak expression in Razek shoots at 5 µM Cd and at slightly lower levels in Chili. At 50 µM Cd, expression remained high in Razek but declined significantly in Chili, suggesting downregulation under acute stress in the latter. This pattern was mirrored by the prooxidant gene *NOX* and the stress-related genes *HSP70* and *ATG8*, which also showed lower expression in Chili under 50 µM Cd. Notably, the small heat shock protein gene *HSP17.6*, typically upregulated by metal stress, was strongly induced in Razek but only moderately in Chili.

Genes of the ASA–GSH cycle, *GR* and *DHAR1*—required to maintain the antioxidant pool under stress—were similarly upregulated in both cultivars at 5 µM Cd. However, *DHAR1* expression continued to rise with increasing Cd concentration, albeit to a lesser extent in Chili at 50 µM Cd. Genes involved in thiol synthesis also showed cultivar-specific responses: *GSH* synthase expression, involved in the synthesis of GSH, increased similarly in both cultivars at 5 µM Cd but declined in Chili at 50 µM Cd. *PCS1*, involved in PC biosynthesis, was induced in both cultivars at 5 µM Cd, although more strongly in Razek, and remained elevated in Razek at 50 µM Cd, while it fell below control levels in Chili.

Cd also induced expression of ABA-responsive genes in both cultivars, but levels were consistently higher in Razek, supporting the general trend of greater stress-related gene activation in Razek under high Cd. These downregulation patterns under acute stress caused by high doses of Cd were confirmed in the time-course experiment, where prolonged exposure to 50 µM Cd led to progressive downregulation of most antioxidant, stress-response, and ABA-signaling genes in Chili (Supplementary Table S6).

## 3. Discussion

A common initial criterion for selecting Cd-tolerant cultivars is the evaluation of seedling biomass or organ length under metal exposure. However, these metrics can yield heterogeneous results and may not fully reflect the capacity to mitigate oxidative stress induced by toxic metals, as observed after *Medicago truncatula* cultivar screening [32]. Differences in growth may also be linked to inherent genotypic features rather than true tolerance mechanisms, as observed in two different *Triticum aestivum* cultivars under Cd stress [33]. In our study, Razek and Chili exhibited distinct responses to Cd stress. Relative variations in seedling radicle elongation and biomass under hydroponic conditions revealed significantly lower growth inhibition in Razek (Figure 1). These differences extended to water uptake, where Razek showed a smaller decline compared to Chili (Figure 2A), indicating better maintenance of water balance. This aligns with previous reports of Cd rapidly impairing water absorption in other species such as pea [34], rice [35], and *Olea europaea* [36], where Cd induced the expression of drought-related genes. In contrast, other general toxicity symptoms, such as lipid peroxidation and chlorophyll loss, followed a typical Cd dose-dependent trend in both cultivars (Figure 2), as known from the literature [6,7,37].

Cd-induced oxidative stress was evident in both cultivars, with differences in antioxidant enzyme responses depending on the organ, as previously reported for Cd-stressed wheat [37,38,39,40,41]. Enzymes involved in H_2_O_2_ detoxification, particularly CAT and APX, were more strongly induced in Chili (Figure 3), suggesting greater oxidative pressure in this cultivar. This trend is consistent with previous studies on Cd-stressed wheat [39], and the importance of CAT under Cd stress is further supported by its role in limiting damage in CAT-deficient tobacco [42]. In this sense, the higher levels of CAT activity occurring in Chili suggest that this cultivar was suffering from stronger Cd stress compared with Razek. The comparison of two different winter wheat cultivars subjected to Cd stress showed a similar pattern, in some cases with hormetic responses of CAT and APX under acute Cd stress [38]. Interestingly, CAT protein levels only increased modestly in shoots, whereas they increased remarkably in roots in both cultivars under Cd stress (Figure 3). CAT transcription peaked at moderate Cd doses before declining at higher concentrations, consistent with transcriptional patterns reported for the CAT gene family [43]. H_2_O_2_ is generated by the action of the ubiquitous family of SOD enzymes that remove O_2_^•―^ accumulated during oxidative stress, of which the CuZnSOD subfamily is the most abundant and widely distributed in plant cells [44]. Also, CuZnSOD showed increased activity, especially in Chili (Figure 3), supported by the gene expression data (Figure 6), as found in wheat [40] or tomato [45]. This pattern matched the overexpression of the *CuZnSOD* gene in response to 5 µM Cd, which remained at high values only in Razek in the presence of 50 µM Cd (Figure 6). *SOD* genes are also overexpressed under Cd stress in wheat plants [46], although downregulation was seen in other species, such as *Arabidopsis* [6] and pea [47].

O_2_^•―^ can be readily generated in plant cells at the apoplast via plasma-membrane-associated NADPH oxidases or internally associated to various electron transport chains working in chloroplasts and mitochondria [7]. In our case, NADPH oxidase was activated only under moderate (5 µM Cd) stress, particularly in Razek (Figure 3). However, under acute stress (50 µM Cd), despite NADPH oxidase gene expression being induced, its activity returned to control levels, particularly in Razek shoots (Figure 6).

Cadmium often disrupts plasma membrane integrity, and under severe cellular damage caused by heavy metals—such as in *Arabidopsis* plants with limited GSH availability—NADPH oxidase activity is markedly inhibited [48], despite continued overexpression of NADPH oxidase genes under Cd stress in wheat [46]. This suggests that ROS production under acute stress by Cd may involve mitochondrial sources rather than membrane-bound oxidases, particularly in non-photosynthetic tissues [49].

To further assess Cd-induced damage in both durum wheat cultivars, we analyzed the accumulation of stress-related proteins such as HSPs and ATG8 (Figure 5). HSPs accumulate under heavy metal stress, usually under acute and short-term shock exposure experiments (i.e., doses exceeding 20 µM Cd) [20,23,24]. In fact, overexpression of regulatory heat shock factors improved the tolerance of wheat and rice to Cd [50]. HSPs (HSP17.6, HSP17.7, and HSP70) accumulated more in Chili roots than in Razek (Figure 4), suggesting greater stress in the former. Although gene expression of *HSP70* and *HSP17.6* was initially induced at moderate Cd levels, it declined in Chili under high Cd stress, indicating possible post-transcriptional regulation that requires further analysis [24]. This was supported by time-course data showing downregulation of *HSP17.6* during prolonged Cd exposure (Supplementary Table S6). Similarly, the autophagy marker ATG8, an important component for the recycling of stress-damaged cellular components [26], did not show strong changes at the protein level but exhibited transient upregulation at 5 µM Cd and sharp downregulation in Chili at 50 µM Cd (Figure 4 and Figure 6). This pattern was also observed upon prolonged Cd exposure (Supplementary Table S6), highlighting the complex regulation of autophagy under heavy metal stress, which implies various stress-related phytohormones and probably several post-translational regulatory mechanisms [51].

Histochemical staining in roots confirmed cultivar-specific ROS and lignin responses to Cd. H_2_O_2_ accumulation, higher in Chili roots, was accompanied by a strong CAT and APX induction (Figure 5), as reported in pea and *Arabidopsis* after short periods (up to 24 h) of Cd and Hg stress that were accompanied with the induction of APX [20,52]. Similarly, very short (0.5 to 3 h) exposure to Cd and other heavy metals resulted in the quick accumulation of H_2_O_2_ in barley root tips, apparently being saturated at concentrations above 20 µM after 2 h treatment [53]. On the contrary, O_2_^•―^ staining declined with increasing Cd dose (Figure 5) and exposure time (Supplementary Figure S3), whereas various reports presented minimal accumulation in *Vicia sativa* [12] and barley root tips [53]. Nevertheless, these results are consistent with the slightly increased CuZnSOD activity that may prevent O_2_^•―^ accumulation, as was previously reported in tobacco leaves [42].

Elevated H_2_O_2_ upon Cd exposure was associated with lignin deposition, particularly in Chili, likely mediated by apoplastic ROS and peroxidase and/or laccase activities, as shown in soybean [13,54]. Similarly, Cd-induced oxidative stress led to lignin biosynthesis in *Brassica parachinensis* [14], and such lignification can alter cell wall properties, promoting premature xylogenesis and contributing to growth inhibition under stress, as shown in barley [55] or rice [56]. Therefore, it is feasible that the stronger lignification observed in Chili may account, at least partially, for the greater inhibition of root growth in this cultivar compared with Razek.

Biothiols are key metabolites for maintaining cellular redox balance in the presence of heavy metals [17]. Biothiol metabolism also showed marked differences between cultivars. GSH levels dropped sharply in Chili under high Cd (Table 1), possibly due to its use as a precursor for PC synthesis [57]. The GSH pool can be replenished by the activation of its synthesis pathway, including the overexpression of GSH synthesis genes and/or activation of GR activity that converts oxidized GSH (GSSG) to GSH [6]. While Razek maintained a high expression of the GSH synthetase gene at 50 µM Cd, its expression declined in Chili, consistent with the time-course data on gene expression (Supplementary Table S4). Inadequate GSH levels may explain the stronger stress symptoms in Chili, as GSH is crucial for detoxification and redox buffering [48,58]. This was partially compensated by increased GR activity in Chili shoots, possibly as a feedback response to restore GSH pools [37]. PCS1 expression and PC2/PC3 accumulation also differed: while both cultivars accumulated PCs (Table 1), Chili showed lower PCS1 expression (Figure 6), suggesting regulation at the post-transcriptional level [59].

Finally, ABA-responsive gene expression increased in both cultivars under Cd but was consistently higher in Razek (Figure 6), reinforcing the view that Razek activates a more robust stress response under acute Cd exposure. Cd-induced modulation of phytohormone signaling, including ABA, ethylene, and jasmonic acid, has been widely reported [35,60]. These phytohormones have complex regulatory mechanisms at different levels, are usually overwhelmed above a certain threshold of cell damage [61], and their interplay with ROS [62] may be critical in shaping cultivar-specific tolerance strategies.

Razek exhibited greater tolerance to Cd stress than Chili, as reflected by more stable water uptake, milder oxidative symptoms, and a more sustained antioxidant and stress-related transcriptional response. In contrast, Chili displayed stronger physiological impairment, with enhanced H_2_O_2_ accumulation, marked GSH depletion, and increased lignin deposition, which may collectively contribute to root growth restriction through oxidative imbalance and stress-induced cell wall remodeling. Overall, these results suggest that cultivar-dependent Cd tolerance in durum wheat relies on the coordinated interplay between ROS control, biothiol-based detoxification, and stress-responsive signaling. Notably, the pronounced downregulation of stress-related genes in Chili at 50 µM Cd occurred concomitantly with GSH depletion and oxidative damage, supporting the interpretation that this response is more consistent with an inability to maintain cellular homeostasis under severe stress rather than an effective acclimation mechanism. Future studies based on time-course analyses, additional biological replication, and targeted assessment of biothiol metabolism, Cd sequestration, and stress/hormonal signaling will be required to clarify the regulatory basis of this transcriptional response and its contribution to Cd sensitivity.

## 4. Materials and Methods

### 4.1. Plant Material and Growth Conditions

Chili and Razek durum wheat (*Triticum turgidum* ssp. *durum*) cultivars were selected for their contrasting responses to Cd stress, and seeds were obtained from the National Institute of *Coopérative Centrale des Grandes Cultures* (CCGC), Tunisia. Kernels were surface sterilized and germinated on moist filter paper in Petri dishes for 72 h. After germination, the seedlings (6–8 per holder) were transferred to an aerated hydroponic system mounted on glass cylinders (300 mL) (Supplementary Figure S2) containing a modified Hoagland nutrient medium (Supplementary Table S1). Plants were grown in a growth chamber with controlled environmental conditions: PAR light averaging 150 μmol·m^−2^ s^−1^, a long-day photoperiod (16 h light/8 h dark), and 25/18 °C day/night temperatures. After 18 d of growth, Cd (as Cd^2+^) (CdCl_2_) was added at different doses (0 (control), 5, and 50 μM) for 72 h. In addition, a second experiment was performed to follow up on the physiological parameters of Chili to 50 µM Cd after 0, 24, 48, and 96 h of exposure using the same experimental setup.

### 4.2. Water Uptake, Total Chlorophyll Content, and Lipid Peroxidation

H_2_O uptake/transpiration was measured from the amount of water replaced in the Hoagland nutrient solution every 24 h from the start of the Cd treatments. Total chlorophyll content was quantified colorimetrically after extraction of 50 mg of leaf tissue with acetone 80% (*v*/*v*), measuring absorbances at 663 and 645 nm. Lipid peroxidation was estimated in 100 mg of sample by analyzing the concentration of the by-product malondialdehyde using a previously described method based on its colorimetric reaction with thiobarbituric acid [52].

### 4.3. Cadmium Concentration

Sampled shoots and roots were dried at 75 °C for 72 h, and the dried material was ground using a mortar and pestle. A total of 100 mg of the sample was acid digested (HNO_3_:H_2_O_2_:H_2_O, 0.3:0.2:0.5, *v*/*v*) using an autoclave (Presoclave-75 Selecta Autoclave, Barcelona, Spain) at 1.5 atm, 120 °C, for 30 min. Digests were filtered and diluted to a final volume of 5 mL with MiliQ water. Cd concentration was determined by ICP-MS (Perkin-Elmer SciexNexION 300, San Jose, CA, USA) [52].

### 4.4. Protein Extraction and Immunodetection

Protein extraction was prepared by grinding 0.5 g of frozen sample in 1 mL of freshly prepared extraction solution (30 mM MOPS at pH 7.5 mM Na_2_-EDTA, 10 mM DTT, 10 mM ascorbic acid, 0.6% PVP, 100 µM PMSF, and protease inhibitor cocktail (P2714, Sigma–Aldrich, St. Louis, MO, USA)), supplemented with 100 mg of polyvinylpolypyrrolidone (PVPP) and centrifuged at 13,000× *g* for 15 min at 4 °C. The supernatant was separated into single-use aliquots, frozen in liquid N_2_, and stored at −80 °C. Protein concentration was determined using the Protein Assay Reagent (BioRad, Hercules, CA, USA) and BSA as standard. For fine protein loading adjustment, extracts were separated using denaturing polyacrylamide (10%) gel electrophoresis (SDS-PAGE), and protein bands were visualized after Coomassie Blue staining [63]. Immunodetection was performed by Western blot after separation of 20 μg of protein, followed by protein electro-blotting onto a nitrocellulose membrane (BioTrace-NT Pall Corporation, East Hills, NY, USA) using a conventional wet transfer procedure. The membrane was blocked with 5% fat-free milk in Tris buffer–saline solution and incubated overnight at 4 °C with the primary antibodies (diluted 1/1000) α-ATG8 (AS07 256), α-GR (AS06 181), α-CAT (AS152 991), α-APX (AS06 180), α-HSP17.6 (AS08 372), α-SHSP17.7 (AS07 255), and α-HSP70 (AS08 371) (Agrisera, Vännäs, Sweden). Membranes were incubated with the secondary antibody (Goat α-rabbit IgG-HRP antibody AS09 602; Agrisera). Specific protein bands were detected using the Lumi-Sensor-Chemiluminescent HRP Substrate Kit (GenScript, Piscataway, NJ, USA), and analyzed using a ChemiDocTM XRS+ System (BioRad, Hercules, CA, USA).

### 4.5. Redox Enzymatic Activities

Protein extracts were mixed with Laemmli buffer without SDS, and 10 to 25 µg protein per sample—depending on the enzymatic assay—was loaded on non-denaturing polyacrylamide gels and separated by electrophoresis (ND-PAGE). Gels were incubated using specific staining procedures as follows: **APX** activity was detected by reverse staining, based on the ascorbate-dependent reduction of nitroblue tetrazolium (NBT) [64]. **SOD** isoforms were detected based on their ability to inhibit the reduction of NBT by O_2_^●−^ [31]. **GR** activity was visualized through the NADPH-dependent reduction of GSSG, using tetrazolium bromide and 2,6-dichlorophenol indophenol as redox indicators [48]. **NADPH oxidase** activity was visualized by the reduction of NBT by O_2_^●−^ generated from NADPH in the presence of CaCl_2_ and MgCl_2_ [65]. **CAT** activity was determined based on its reaction with H_2_O_2_ in the presence of K_3_Fe(CN)_6_ and FeCl_3_ [66]. Gels were photographed using a ChemiDocTM XRS+ System (BioRad).

### 4.6. Analysis of Biothiols

The biothiol concentration was determined by HPLC [52] using 100 mg of frozen plant material that was homogenized in 300 μL of 0.25M HCl in the presence of 50 mg of PVPP, and N-acetyl cysteine was added as internal standard (25 μM final concentration). After centrifugation (12,000× *g*, 15 min at 4 °C), 100 μL of the sample was injected into a Mediterranean SEA18 column (250 mm × 4.6 mm; Teknokroma, Sant Cugat del Vallés, Spain) using an Agilent 1200 HPLC-DAD system (Santa Clara, CA, USA). Biothiols were detected by a post-column reaction with Ellman’s, and the absorbance was recorded at 412 nm.

### 4.7. Cellular Detection of ROS and Lignin

Superoxide ions (**O_2_^●−^**) were visualized in wheat roots using 1.25 mM NBT dissolved in 50 mM NaH_2_PO_4_ buffer (pH 7.8). Roots were immersed in the solution, then kept in the dark until sufficient formazan precipitate appeared. Root sections were prepared using a sharp razorblade, and observed under an Olympus IX-70 S22 inverted microscope (Hachioji, Tokyo, Japan). Extracellular **H_2_O_2_** was detected in root segments (1 cm long) by AmplexRed fluorescence [20]. Segments were firstly equilibrated in 2 mM MES pH 6.0 for 1 h, then placed in 96-well black plates with 200 μL of the same buffer. Next, 10 μL of peroxidase (0.05 mg/mL) was added, and fluorescence was read using a BioTek Synergy HT (Winooski, VT, USA) plate reader (λ_exc_ = 542 nm, λ_em_ = 590 nm) every 5 min for 90 min. **Lignin** was visualized in roots using phloroglucinol, prepared by mixing freshly made phloroglucinol reagent (0.3 g in 10 mL ethanol), 1 mL of concentrated HCl, and 1 mL of water (1:1:1 (*v*/*v*/*v*)). After 10 min incubation, root segments were rinsed with distilled water, cut with a sharp razorblade and examined under an Olympus IX-70 S22 inverted microscope.

### 4.8. Gene Expression Analysis

Gene expression was analyzed by quantitative RT-PCR (qPCR) using 50 to 100 mg of a −80 °C frozen shoot sample. This was pulverized in liquid N_2_ using a mortar and pestle, and the powder was suspended in Pellet Solubilization Buffer (PSB) (7 M guanidine–HCl, 2% (*v*/*v*) Tween 20, 4% (*v*/*v*) Nonidet P-40, 50 mM Tris–HCl at pH 7.5, supplemented with 1% (*v*/*v*) ß-mercaptoethanol), adjusting PSB to a 8 µL/mg sample ratio [67]. Using a silica column to retain nucleic acids (EconoSpin Cat. No 1920-250, Epoch Life Sciences, Missouri City, TX, USA), RNA was prepared after various steps and incubations, which are detailed in the Extended Materials and Methods (Supplementary Materials). DNA was removed using the TURBO DNA-free kit (Ambion, Austin, TX, USA). RNA concentration and purity were determined with a NanoDrop^®^ ND-1000 spectrophotometer (Thermo Fisher Scientific, Waltham, MA, USA). For cDNA synthesis, 5 µg of RNA input was reverse-transcribed using the PrimeScript™ RT Reagent Kit (Takara Bio Inc., Kusatsu, Japan), according to the manufacturer’s instructions. The resulting cDNA was diluted 1/10 in TE buffer (1 mM Tris–HCl, 0.1 mM EDTA, pH 8.0) and stored at −20 °C until further analysis. Quantitative real-time PCR (qPCR) was conducted using the 7500 Fast Real-Time PCR System (Applied Biosystems, Foster City, CA, USA) with 2 µL of cDNA, 5 µL of QuantiNova SYBR^®^ Green PCR Master Mix (Qiagen, Venlo, The Netherlands), 0.05 µL of QN ROX Reference Dye, 2.6 µL of RNase-free H_2_O, and 0.3 µL of each pair of primers (sequences available in Supplementary Table S2). The cycling protocol included 2 min at 95 °C, followed by 60 cycles of 95 °C for 5 s, and an extension at 60 °C for 10 s. A dissociation curve was generated to confirm amplicon specificity. Relative gene expression levels were calculated using the 2^−ΔCq^ method, with normalization based on the GrayNorm algorithm [67] using up to five reference genes (see Supplementary Table S2).

### 4.9. Statistical Analysis

Data are presented as mean ± standard deviation (S.D.) of independent biological replicates. Statistical analyses were performed using IBM SPSS Statistics software (version 22). Due to the experimental design, in which the two cultivars were grown in separate hydroponic containers (independent treatment blocks), statistical comparisons were performed within each cultivar across Cd treatments using one-way analysis of variance (ANOVA), followed by Duncan’s multiple range test for post hoc comparisons. Differences were considered statistically significant at *p* < 0.05. Prior to ANOVA, data distribution was checked using Shapiro–Wilk and Kolmogorov–Smirnov tests to verify normality.

## 5. Conclusions

This pilot hydroponic study shows that the two durum wheat cultivars, Razek and Chili, display contrasting physiological and molecular responses to cadmium (Cd) stress. Although Chili generally exhibited higher absolute growth-related traits under control conditions, Cd exposure caused a stronger relative inhibition of root elongation, biomass accumulation, and water uptake in this cultivar compared with Razek. Overall, Razek maintained a better water status and exhibited lower oxidative damage, together with more stable antioxidant capacity and the preservation or induction of key stress-related genes under Cd exposure. In contrast, Chili showed pronounced sensitivity, characterized by stronger H_2_O_2_ accumulation, enhanced lignin deposition, depletion of GSH, and downregulation of genes involved in redox regulation and stress signaling.

Taken together, these results support a working model in which Razek tolerance reflects a coordinated response combining more efficient ROS control, improved biothiol-based buffering/detoxification, and better integration of stress signaling, which collectively limit secondary constraints, such as water uptake impairment and stress-induced lignification. Conversely, the stronger oxidative imbalance and lignification observed in Chili may contribute to root growth restriction under Cd. While these findings identify Razek as a promising genotype for further investigation, confirmation of its agronomic suitability will require soil- and field-based studies, including analyses of Cd partitioning/sequestration, hormonal regulation (e.g., ABA-related responses), and the kinetics of cell wall remodeling during early Cd exposure. Importantly, from a toxicological perspective, future work should also quantify Cd translocation and accumulation in kernels to evaluate potential food-safety implications and guide safe agronomic practices.

## Figures and Tables

**Table 1:** Biothiol concentration (nmol/gFW): cysteine (Cys), glutathione (GSH), phytochelatin 2 (PC2), and phytochelatin 3 (PC3) in shoots and roots of Razek and Chili durum wheat under Cd (0, 5, and 50 µM) for 72 h. Mean ± S.D. of three biological replicates; green/red indicate significant up/down variation relative to the control. Asterisks show differences between varieties (*p* < 0.05; one-way ANOVA).

		Shoot		Root	
		Razek	Chili	Razek	Chili
**Cys**	Control	54.4 ± 3.1	47.4 ± 3.3	78.8 ± 11.5	71.9 ± 11.3
	5 µM Cd	53.5 ± 5.1	46.6 ± 1.7	66.23 ± 9.3	58.2 ± 9.1
	50µM Cd	47.9 ± 2.8	43.6 ± 1.3	82.4 ± 9.0	82.4 ± 9.0
**GSH**	Control	219.9 ± 30.6	248.1 ± 12.6	91.7 ± 9.4	91.93 ± 6.8
	5 µM Cd	243.0 ± 11.9	288.9 ± 6.8	140.4 ± 32.4	109.2 ± 3.4
	50µM Cd	369.6 ± 4.7 *	234.1 ± 16.5 *	204.6 ± 77.1 *	60.7 ± 1.0 *
**PC2**	Control	n.d.	n.d.	n.d.	n.d.
	5 µM Cd	n.q.	n.q.	61.4 ± 3.7	56.3 ± 7.1
	50µM Cd	38.7 ± 0.9	43.3 ± 3.6	86.9 ± 12.0	80.81 ± 17.4
**PC3**	Control	n.d.	n.d.	n.d.	n.d.
	5 µM Cd	n.q.	n.q.	77.2 ± 19.8 *	41.8 ± 3.6 *
	50µM Cd	52.7 ± 14.5	54.1 ± 3.2	105.5 ± 15.0	106.7 ± 9.0

n.d. Not detected. n.q.: Not quantifiable.

## Data Availability

The data presented in this study will be openly available, upon acceptance of the manuscript, in the institutional repository of the Universidad Autónoma de Madrid at a persistent URL.

## Supplementary Materials

The following supporting information can be downloaded at https://www.mdpi.com/article/10.3390/plants15030418/s1, Supplementary Materials provide additional details on the experimental procedures and data, as follows: Table S1. Composition of Hoagland’s nutrient solution. Table S2. Primers used for the qRT-PCR analysis. Table S3. Absolute biometric values of Razek and Chili plants after 72 h of exposure to 0 (control), 5 and 50 µM Cd. Table S4. Concentration of biothiols in Cd-treated Chili plants in the time-course complementary experiment, with plants exposed to 50 µM Cd for 0, 24, 48, and 96 h. Table S5. Gene expression data with statistical significance and standard deviation corresponding to Figure 6. Table S6. Gene expression data with statistical significance and standard deviation corresponding to Chili plants exposed to Cd in the time-course complementary experiment. Figure S1. Images of durum wheat kernels in the seed germination assay used for the Cd tolerance screening tests. Figure S2. Pictures of the hydroponic system used to grow durum wheat seedlings to analyze the physiological, biochemical and molecular responses to Cd. Figure S3. (A) Antioxidant activities and stress proteins of Chili seedlings treated with Cd in the time-course complementary experiment. (B) O_2_^•―^ and lignin histochemical staining of Chili seedlings in the same time-course complementary experiment.

## References

[1] Annabi A., Said K. (2013). Cadmium: Bioaccumulation, Histopathology and Detoxifying Mechanisms in Fish. Am. J. Res. Commun..

[2] Abdelwaheb M., Nedeff V., Dridi-Dhaouadi S., Moșneguțu E., Barsan N., Chițimus A.-D. (2022). Assessment of Cadmium and Copper Adsorption by Two Agricultural Soils from Romania and Tunisia: Risk of Water Resource Pollution. Processes.

[3] Tóth G., Hermann T., Szatmári G., Pásztor L. (2016). Maps of Heavy Metals in the Soils of the European Union and Proposed Priority Areas for Detailed Assessment. Sci. Total Environ..

[4] Mei S., Lin K., Williams D.V., Liu Y., Dai H., Cao F. (2022). Cadmium Accumulation in Cereal Crops and Tobacco: A Review. Agronomy.

[5] Clemens S., Ma J.F. (2016). Toxic Heavy Metal and Metalloid Accumulation in Crop Plants and Foods. Annu. Rev. Plant Biol..

[6] Jozefczak M., Bohler S., Schat H., Horemans N., Guisez Y., Remans T., Vangronsveld J., Cuypers A. (2015). Both the Concentration and Redox State of Glutathione and Ascorbate Influence the Sensitivity of Arabidopsis to Cadmium. Ann. Bot..

[7] Cuypers A., Vanbuel I., Iven V., Kunnen K., Vandionant S., Huybrechts M., Hendrix S. (2023). Cadmium-Induced Oxidative Stress Responses and Acclimation in Plants Require Fine-Tuning of Redox Biology at Subcellular Level. Free Radic. Biol. Med..

[8] Hafsi C., Collado-Arenal A.M., Wang H., Sanz-Fernández M., Sahrawy M., Shabala S., Romero-Puertas M.C., Sandalio L.M. (2022). The Role of NADPH Oxidases in Regulating Leaf Gas Exchange and Ion Homeostasis in *Arabidopsis* Plants under Cadmium Stress. J. Hazard Mater..

[9] Foyer C.H., Noctor G. (2003). Redox Sensing and Signalling Associated with Reactive Oxygen in Chloroplasts, Peroxisomes and Mitochondria. Physiol. Plant..

[10] Foyer C.H., Noctor G. (2011). Ascorbate and Glutathione: The Heart of the Redox Hub. Plant Physiol..

[11] Anjum N.A., Sharma P., Gill S.S., Hasanuzzaman M., Khan E.A., Kachhap K., Mohamed A.A., Thangavel P., Devi G.D., Vasudhevan P. (2016). Catalase and Ascorbate Peroxidase—Representative H2O2-Detoxifying Heme Enzymes in Plants. Environ. Sci. Pollut. Res..

[12] Rui H., Chen C., Zhang X., Shen Z., Zhang F. (2016). Cd-Induced Oxidative Stress and Lignification in the Roots of Two *Vicia sativa* L. Varieties with Different Cd Tolerances. J. Hazard Mater..

[13] Yang Y.J., Cheng L.M., Liu Z.H. (2007). Rapid Effect of Cadmium on Lignin Biosynthesis in Soybean Roots. Plant Sci..

[14] Wang X.-S., Yang Q.-L., Peng M.-J., He C.-T. (2022). Simultaneous Elevation of Acid-Insoluble Lignin and Syringyl Lignin Is the Preliminary Cd Detoxification Strategy in a Cd Pollution-Safe Cultivar (Cd-PSC) of *Brassica parachinensis* L. Environ. Exp. Bot..

[15] Dalal M., Sahu S., Tiwari S., Rao A.R., Gaikwad K. (2018). Transcriptome Analysis Reveals Interplay between Hormones, ROS Metabolism and Cell Wall Biosynthesis for Drought-Induced Root Growth in Wheat. Plant Physiol. Biochem..

[16] Cobbett C.S. (2000). Phytochelatins and Their Roles in Heavy Metal Detoxification. Plant Physiol..

[17] Iven V., Vanbuel I., Hendrix S., Cuypers A. (2023). The Glutathione-Dependent Alarm Triggers Signalling Responses Involved in Plant Acclimation to Cadmium. J. Exp. Bot..

[18] Serrano N., Díaz-Cruz J.M., Ariño C., Esteban M. (2015). Recent Contributions to the Study of Phytochelatins with an Analytical Approach. TrAC Trends Anal. Chem..

[19] Song W.Y., Mendoza-Cózatl D.G., Lee Y., Schroeder J.I., Ahn S.N., Lee H.S., Wicker T., Martinoia E. (2014). Phytochelatin-Metal(Loid) Transport into Vacuoles Shows Different Substrate Preferences in Barley and Arabidopsis. Plant Cell Environ..

[20] Flores-Cáceres M.L., Ortega-Villasante C., Carril P., Sobrino-Plata J., Hernández L.E. (2023). The Early Oxidative Stress Induced by Mercury and Cadmium Is Modulated by Ethylene in *Medicago sativa* Seedlings. Antioxidants.

[21] Wu J., Gao T., Hu J., Zhao L., Yu C., Ma F. (2022). Research Advances in Function and Regulation Mechanisms of Plant Small Heat Shock Proteins (SHSPs) under Environmental Stresses. Sci. Total Environ..

[22] Hsu Y.T., Kao C.H. (2007). Heat Shock-Mediated H_2_O_2_ Accumulation and Protection against Cd Toxicity in Rice Seedlings. Plant Soil.

[23] Yang Y., Li X., Yang S., Zhou Y., Dong C., Ren J., Sun X., Yang Y. (2015). Comparative Physiological and Proteomic Analysis Reveals the Leaf Response to Cadmium-Induced Stress in Poplar (*Populus yunnanensis*). PLoS ONE.

[24] Kochhar S., Kochhar V.K. (2005). Expression of Antioxidant Enzymes and Heat Shock Proteins in Relation to Combined Stress of Cadmium and Heat in *Vigna mungo* Seedlings. Plant Sci..

[25] Weber M., Trampczynska A., Clemens S. (2006). Comparative Transcriptome Analysis of Toxic Metal Responses in *Arabidopsis thaliana* and the Cd^2+^-Hypertolerant Facultative Metallophyte *Arabidopsis halleri*. Plant Cell Environ..

[26] Avin-Wittenberg T. (2019). Autophagy and Its Role in Plant Abiotic Stress Management. Plant Cell Environ..

[27] Doelling J.H., Walker J.M., Friedman E.M., Thompson A.R., Vierstra R.D. (2002). The APG8/12-Activating Enzyme APG7 Is Required for Proper Nutrient Recycling and Senescence in *Arabidopsis thaliana*. J. Biol. Chem..

[28] Michaeli S., Galili G., Genschik P., Fernie A.R., Avin-Wittenberg T. (2016). Autophagy in Plants—What’s New on the Menu?. Trends Plant Sci..

[29] Signorelli S., Tarkowski Ł.P., Van den Ende W., Bassham D.C. (2019). Linking Autophagy to Abiotic and Biotic Stress Responses. Trends Plant Sci..

[30] Zhou S., Han Y.-Y., Chen Y., Kong X., Wang W. (2015). The Involvement of Expansins in Response to Water Stress during Leaf Development in Wheat. J. Plant Physiol..

[31] Beauchamp C., Fridovich I. (1971). Superoxide Dismutase: Improved Assays and an Assay Applicable to Acrylamide Gels. Anal. Biochem..

[32] García de la Torre V.S., Coba de la Peña T., Lucas M.M., Pueyo J.J. (2013). Rapid Screening of *Medicago truncatula* Germplasm for Mercury Tolerance at the Seedling Stage. Environ. Exp. Bot..

[33] Yotsova E., Dobrikova A., Stefanov M., Misheva S., Bardáčová M., Matušíková I., Žideková L., Blehová A., Apostolova E. (2020). Effects of Cadmium on Two Wheat Cultivars Depending on Different Nitrogen Supply. Plant Physiol. Biochem..

[34] Hernández L.E., Gárate A., Carpena-Ruiz R. (1997). Effects of Cadmium on the Uptake, Distribution and Assimilation of Nitrate in *Pisum sativum*. Plant Soil.

[35] Oono Y., Yazawa T., Kawahara Y., Kanamori H., Kobayashi F., Sasaki H., Mori S., Wu J., Handa H., Itoh T. (2014). Genome-Wide Transcriptome Analysis Reveals That Cadmium Stress Signaling Controls the Expression of Genes in Drought Stress Signal Pathways in Rice. PLoS ONE.

[36] Zouari M., Ben Ahmed C., Elloumi N., Bellassoued K., Delmail D., Labrousse P., Ben Abdallah F., Ben Rouina B. (2016). Impact of Proline Application on Cadmium Accumulation, Mineral Nutrition and Enzymatic Antioxidant Defense System of *Olea europaea L.* Cv Chemlali Exposed to Cadmium Stress. Ecotoxicol. Environ. Saf..

[37] Yannarelli G.G., Fernández-Alvarez A.J., Santa-Cruz D.M., Tomaro M.L. (2007). Glutathione Reductase Activity and Isoforms in Leaves and Roots of Wheat Plants Subjected to Cadmium Stress. Phytochemistry.

[38] Milone M.T., Sgherri C., Clijsters H., Navari-Izzo F. (2003). Antioxidative Responses of Wheat Treated with Realistic Concentration of Cadmium. Environ. Exp. Bot..

[39] Ozfidan-Konakci C., Yildiztugay E., Bahtiyar M., Kucukoduk M. (2018). The Humic Acid-Induced Changes in the Water Status, Chlorophyll Fluorescence and Antioxidant Defense Systems of Wheat Leaves with Cadmium Stress. Ecotoxicol. Environ. Saf..

[40] Guo J., Qin S., Rengel Z., Gao W., Nie Z., Liu H., Li C., Zhao P. (2019). Cadmium Stress Increases Antioxidant Enzyme Activities and Decreases Endogenous Hormone Concentrations More in Cd-Tolerant than Cd-Sensitive Wheat Varieties. Ecotoxicol. Environ. Saf..

[41] Kaya C., Ashraf M., Alyemeni M.N., Ahmad P. (2020). Responses of Nitric Oxide and Hydrogen Sulfide in Regulating Oxidative Defence System in Wheat Plants Grown under Cadmium Stress. Physiol. Plant..

[42] Iannone M.F., Groppa M.D., Benavides M.P. (2015). Cadmium Induces Different Biochemical Responses in Wild Type and Catalase-Deficient Tobacco Plants. Environ. Exp. Bot..

[43] Ghorbel M., Zribi I., Besbes M., Bouali N., Brini F. (2023). Catalase Gene Family in Durum Wheat: Genome-Wide Analysis and Expression Profiling in Response to Multiple Abiotic Stress Conditions. Plants.

[44] Alscher R.G., Erturk N., Heath L.S. (2002). Role of Superoxide Dismutases (SODs) in Controlling Oxidative Stress in Plants. J. Exp. Bot..

[45] Nogueirol R.C., Monteiro F.A., Gratão P.L., de Alcântara da Silva B.K., Azevedo R.A. (2016). Cadmium Application in Tomato: Nutritional Imbalance and Oxidative Stress. Water Air Soil Pollut..

[46] Zhang T., Xiao J., Zhao Y., Zhang Y., Jie Y., Shen D., Yue C., Huang J., Hua Y., Zhou T. (2021). Comparative Physiological and Transcriptomic Analyses Reveal Ascorbate and Glutathione Coregulation of Cadmium Toxicity Resistance in Wheat Genotypes. BMC Plant Biol..

[47] Romero-Puertas M.C., Corpas F.J., Rodríguez-Serrano M., Gómez M., del Río L.A., Sandalio L.M. (2007). Differential Expression and Regulation of Antioxidative Enzymes by Cadmium in Pea Plants. J. Plant Physiol..

[48] Sobrino-Plata J., Carrasco-Gil S., Abadía J., Escobar C., Álvarez-Fernández A., Hernández L.E. (2014). The Role of Glutathione in Mercury Tolerance Resembles Its Function under Cadmium Stress in *Arabidopsis*. Metallomics.

[49] Heyno E., Klose C., Krieger-Liszkay A. (2008). Origin of Cadmium-Induced Reactive Oxygen Species Production: Mitochondrial Electron Transfer versus Plasma Membrane NADPH Oxidase. New Phytol..

[50] Shim D., Hwang J.U., Lee J., Lee S., Choi Y., An G., Martinoia E., Lee Y. (2009). Orthologs of the Class A4 Heat Shock Transcription Factor HsfA4a Confer Cadmium Tolerance in Wheat and Rice. Plant Cell.

[51] Wang P., Wang T., Han J., Li M., Zhao Y., Su T., Ma C. (2021). Plant Autophagy: An Intricate Process Controlled by Various Signaling Pathways. Front. Plant Sci..

[52] Sobrino-Plata J., Barón-Sola Á., Ortega-Villasante C., Ortega-Campayo V., González-Berrocal C., Conesa-Quintana C., Carrasco-Gil S., Muñoz-Pinilla M., Abadía J., Álvarez-Fernández A. (2021). Sulphur and Biothiol Metabolism Determine Toxicity Responses and Fate of Mercury in *Arabidopsis*. Environ. Exp. Bot..

[53] Tamás L., Mistrík I., Zelinová V. (2017). Heavy Metal-Induced Reactive Oxygen Species and Cell Death in Barley Root Tip. Environ. Exp. Bot..

[54] Loix C., Huybrechts M., Vangronsveld J., Gielen M., Keunen E., Cuypers A. (2017). Reciprocal Interactions between Cadmium-Induced Cell Wall Responses and Oxidative Stress in Plants. Front. Plant Sci..

[55] Ďurčeková K., Huttová J., Mistrík I., Ollé M., Tamás L. (2007). Cadmium Induces Premature Xylogenesis in Barley Roots. Plant Soil.

[56] Dong Q., Wu Y., Li B., Chen X., Peng L., Sahito Z.A., Li H., Chen Y., Tao Q., Xu Q. (2023). Multiple Insights into Lignin-Mediated Cadmium Detoxification in Rice (*Oryza sativa*). J. Hazard Mater..

[57] Cui W., Chen H., Zhu K., Jin Q., Xie Y., Cui J., Xia Y., Zhang J., Shen W. (2014). Cadmium-Induced Hydrogen Sulfide Synthesis Is Involved in Cadmium Tolerance in *Medicago sativa* by Reestablishment of Reduced (Homo)Glutathione and Reactive Oxygen Species Homeostases. PLoS ONE.

[58] Flores-Cáceres M.L., Hattab S., Hattab S., Boussetta H., Banni M., Hernández L.E. (2015). Specific Mechanisms of Tolerance to Copper and Cadmium Are Compromised by a Limited Concentration of Glutathione in Alfalfa Plants. Plant Sci..

[59] Wu C., Zhang J., Chen M., Liu J., Tang Y. (2024). Characterization of a Nicotiana Tabacum Phytochelatin Synthase 1 and Its Response to Cadmium Stress. Front. Plant Sci..

[60] Gómez-Sagasti M.T., Barrutia O., Ribas G., Garbisu C., Becerril J.M. (2016). Early Transcriptomic Response of: *Arabidopsis thaliana* to Polymetallic Contamination: Implications for the Identification of Potential Biomarkers of Metal Exposure. Metallomics.

[61] Chmielowska-Bak J., Gzyl J., Rucinska-Sobkowiak R., Arasimowicz-Jelonek M., Deckert J. (2014). The New Insights into Cadmium Sensing. Front. Plant Sci..

[62] Xia X.J., Zhou Y.H., Shi K., Zhou J., Foyer C.H., Yu J.Q. (2015). Interplay between Reactive Oxygen Species and Hormones in the Control of Plant Development and Stress Tolerance. J. Exp. Bot..

[63] Laemmli U.K. (1970). Cleavage of Structural Proteins during the Assembly of the Head of Bacteriophage T4. Nature.

[64] Jiménez A., Hernández J.A., Barceló A.R., Sandalio L.M., Del Río L.A., Sevilla F. (1998). Mitochondrial and Peroxisomal Ascorbate Peroxidase of Pea Leaves. Physiol. Plant..

[65] Sagi M., Fluhr R. (2001). Superoxide Production by Plant Homologues of the Gp91phox NADPH Oxidase. Modulation of Activity by Calcium and by Tobacco Mosaic Virus Infection. Plant Physiol..

[66] Woodbury W., Spencer A.K., Stahmann M.A. (1971). An Improved Procedure Using Ferricyanide for Detecting Catalase Isozymes. Anal. Biochem..

[67] Huybrechts M., Hendrix S., Bertels J., Beemster G.T.S., Vandamme D., Cuypers A. (2020). Spatial Analysis of the Rice Leaf Growth Zone under Controlled and Cadmium-Exposed Conditions. Environ. Exp. Bot..

