# Supplementary material for: Characterization of the Differential Tolerance of Two Triticum durum Cultivars to Short-Term Cadmium-Induced Stress
Source: Plants (Basel). 2026 Jan 29;15(3):418. doi: 10.3390/plants15030418 (PMC12899852; doi:10.3390/plants15030418)
Supplement: Supplementary file 1 [file plants-15-00418-s001.zip › plants-4097882-supplementary.pdf]

SUPPLEMENTARY MATERIAL

Extended Materials & Methods

1.1 Protein extraction and immunodetection

Proteins were extracted by grinding 0.5 g of frozen plant material in 1 mL of freshly prepared extraction buffer containing 30 mM MOPS (pH 7.5), 5 mM Na_2-EDTA, 10 mM DTT, 10 mM ascorbic acid, 0.6% (w/v) polyvinylpyrrolidone (PVP), 10 μL of 100 mM PMSF, and 1 mL of protease inhibitor cocktail (P2714, Sigma–Aldrich, St. Louis, MO, USA). Homogenates were centrifuged at 13,000 × *g* for 15 min at 4 °C, and the resulting supernatants were aliquoted and stored at −80 °C until use. Protein concentration was determined using the Bradford method (Bio-Rad Protein Assay Reagent, Hercules, CA, USA), with BSA as the standard. Protein loading for enzyme activity assays and immunodetection was adjusted based on SDS-PAGE followed by Coomassie Brilliant Blue staining [1].

For immunodetection, protein samples were mixed with Laemmli loading buffer and denatured at 90 °C for 10 min. A total of 20 μg of protein was resolved by SDS-PAGE on 10% polyacrylamide gels, using constant voltage (120 V) at 30 mA/gel for 60 min. Proteins were transferred onto nitrocellulose membranes (BioTrace-NT, Pall Corporation, East Hills, NY, USA) using a semi-dry blotting system (Trans-Blot Semi-Dry Transfer Cell, Bio-Rad) for 30 min in transfer buffer (25 mM Tris-HCl, 192 mM glycine, 0.1 % SDS, and 20% methanol, pH 8.3). Membranes were blocked with 5% (w/v) non-fat dry milk in TBS (200 mM Tris-HCl, pH 7.5, 5 M NaCl), and incubated overnight at 4 °C with primary antibodies (1:1000 dilution): α-ATG8 (AS07 256), α-GR (AS06 181), α-CAT (AS152991), α-APX (AS06180), α-HSP17.6 (AS08 372), α-HSP17.7 (AS07 255), and α-HSP70 (AS08 371) (Agrisera, Vännäs, Sweden). After washing with TBS, membranes were incubated for 1 h at room temperature with HRP-conjugated goat anti-rabbit IgG secondary antibody (GenScript, Piscataway, NJ, USA). Specific protein bands were visualized using the LumiSensor Chemiluminescent HRP Substrate Kit (GenScript).

1.2 Redox enzymatic activities

Protein extracts were mixed with Laemmli buffer lacking SDS, and equal volumes containing 10–25 μg of soluble protein (depending on the specific enzymatic assay) were kept on ice until separation by non-denaturing polyacrylamide gel electrophoresis (ND-PAGE), as described by [2]. After electrophoresis, gels were rinsed with deionized water and incubated in specific staining solutions to detect individual antioxidant enzyme activities.

Ascorbate peroxidase (APX) was assessed using 30 μg of protein from shoot and root extracts. Gels were incubated for 30 min in 50 mM potassium phosphate buffer (pH 7.0) containing 4 mM ascorbate, followed by the addition of 2 mM H_2O_2 to initiate the reaction. APX activity bands were visualized by incubating the gels in 50 mM phosphate buffer (pH 7.8) containing 0.5 mM nitroblue tetrazolium (NBT) and 17 mM TEMED. APX activity appears as clear bands on a dark background due to inhibition of NBT reduction [3].

Superoxide dismutase (SOD) was visualized using 30 µg of protein separated on a 12–17% gradient ND-PAGE. Gels were incubated in 50 mM sodium phosphate buffer (pH 7.8) containing 1.25 mM NBT, 0.1 mM EDTA, and 100 µM riboflavin. Gels were then exposed to white light to initiate superoxide production, and SOD activity was observed as achromatic bands where superoxide-driven NBT reduction was blocked [4].

Glutathione reductase (GR) was detected using 30 µg of protein incubated in 50 mM Tris-HCl buffer (pH 7.5) containing 0.5 mM oxidized glutathione (GSSG), 0.14 mM NADPH, and 0.12 mM 3-(4,5-dimethylthiazol-2-yl)-2,5-diphenyltetrazolium bromide (MTT). GR activity reduces GSSG to GSH using NADPH, which in turn reduces MTT to a blue formazan product. A control gel without GSSG was included to confirm staining specificity [5].

NADPH oxidase was evaluated by incubating gels in 50 mM Tris-HCl buffer (pH 7.5) containing 0.1 mM NADPH, 0.1 mM NBT, 1 mM $CaCl_2$, and 1 mM $MgCl_2$. Blue staining indicates superoxide production via NADPH oxidase activity. A control was included with 10 µM diphenyleneiodonium chloride (DPI), a specific NADPH oxidase inhibitor, to validate signal specificity [6].

Catalase (CAT) was assessed by incubating gels in **3.3 mM H_2O_2** for 20 min, followed by staining in a solution containing **1% potassium ferricyanide ($K_3[Fe(CN)_6]$)** and **1% ferric chloride ($FeCl_3$)**. CAT activity appears as clear bands on a dark background due to localized dismutation of H_2O_2 [7].

Gels were photographed using a ChemiDoc™ XRS+ imaging system (Bio-Rad, Hercules, CA, USA)

References

1. Laemmli, U.K. Cleavage of Structural Proteins during the Assembly of the Head of Bacteriophage T4. *Nature* **1970**, doi:10.1038/227680a0.
2. Flores-Cáceres, M.L.; Ortega-Villasante, C.; Carril, P.; Sobrino-Plata, J.; Hernández, L.E. The Early Oxidative Stress Induced by Mercury and Cadmium Is Modulated by Ethylene in *Medicago Sativa* Seedlings. *Antioxidants* **2023**, *12*, 551, doi:10.3390/antiox12030551.
3. Jiménez, A.; Hernández, J.A.; Barceló, A.R.; Sandalio, L.M.; Del Río, L.A.; Sevilla, F. Mitochondrial and Peroxisomal Ascorbate Peroxidase of Pea Leaves. *Physiol. Plant.* **1998**, *104*, 687–692, doi:https://doi.org/10.1034/j.1399-3054.1998.1040424.x.
4. Beauchamp, C.; Fridovich, I. Superoxide Dismutase: Improved Assays and an Assay Applicable to Acrylamide Gels. *Anal. Biochem.* **1971**, *44*, 276–287, doi:https://doi.org/10.1016/0003-2697(71)90370-8.
5. Sobrino-Plata, J.; Carrasco-Gil, S.; Abadía, J.; Escobar, C.; Álvarez-Fernández, A.; Hernández, L.E. The Role of Glutathione in Mercury Tolerance Resembles Its Function under Cadmium Stress in Arabidopsis. *Metallomics* **2014**, *6*, 356, doi:10.1039/c3mt00329a.
6. Sagi, M.; Fluhr, R. Superoxide Production by Plant Homologues of the Gp91phox NADPH Oxidase. Modulation of Activity by Calcium and by Tobacco Mosaic Virus Infection. *Plant Physiol.* **2001**, *126*, 1281–1290, doi:10.1104/pp.126.3.1281.
7. Woodbury, W.; Spencer, A.K.; Stahmann, M.A. An Improved Procedure Using Ferricyanide for Detecting Catalase Isozymes. *Anal. Biochem.* **1971**, *44*, 301–305, doi:10.1016/0003-2697(71)90375-7.

Supplementary Table S1: Composition of the nutrient solution used for culture media (Hoagland's solution).

	Compound	Concentration (M)
Macronutrients	$MsSO4\cdot 7H_2O$<br>$NaCl$	$0.5\cdot 10^{-3}$<br>$0.4\cdot 10^{-3}$
	$Ca(NO_3)_2\cdot 4H_2O$<br>KNO_3<br>$Mg(NO_3)_2\cdot 6H_2O$	$2.0\cdot 10^{-3}$<br>$1.5\cdot 10^{-3}$<br>$1.0\cdot 10^{-3}$
	KH_2PO_4	$1.0\cdot 10^{-3}$
Micronutrients	$MnSO_4$<br>$ZnSO_4$<br>$CuSO_4$<br>$Mo_7O_{24}(NH_4)_6$	$1.8\cdot 10^{-5}$<br>$3.0\cdot 10^{-6}$<br>$6.0\cdot 10^{-6}$<br>$2.0\cdot 10^{-6}$
	H_3BO_3	$25.0\cdot 10^{-6}$
	Fe (EDHHA)<br>Sequestrene (6%)	$45.0\cdot 10^{-6}$

Supplementary Table S2. List of gene-specific primers used for qRT-PCR analysis in our experiments to analyse transcription in wheat (*Triticum turgidum*) under Cd stress.

Gene	Locus	Forward primer (5'-3')	Reverse primer (5'-3')	Amplicon size
**pAPX**	EF555121.1	CCCATGGTTCAAATGCTGGC	CCTTCACGGGGACAAACTGA	187
**GSH1**	AY864064.1	GAGAGGTGCTGATGGTGGAC	CTGGGACCTTCCGTCTCAAC	155
**HSP70**	XM044527774	GGGAGGACTTTGACAACCGT	ATCTCGATGGTGGTTTGGGC	163
**MnSOD**	KP696754.1	CATCAAGTTCAACGGCGGC	CCACACCCATCCAGATCCTT	196
**CAT**	KP696753.1	ACACCTACACGCTGGTGAAC	TGTAGAAGGTCCACTCCGGG	189
**ABA**	KX660744.1	CGAGTACGAGCGGATCACC	CTCGTGCTTCTCGTAGAGGG	109
**ATG8**	XM044467528.1	CGGTGATCGTTGAGAAGGCT	TTGATCCTCTTCCGCACCAC	118
**GR**	XM044506346.1	GAAAAACTGCAGCTGAGGCA	TTCGGCCGGTGGCATC	109
**GPX**	KM817777.1	ACAAGGTTGATGTCAACGGC	ACGTGACCCTCCTTGTCAAC	130
**HSP17,6**	AJ971358.1	CAAGCTGATGCGCAAGTTCG	ATGGTCTTGGGCTTCTTGGG	132
**DHAR1**	XM044599778.1	AGTCTCCAGAACCCGGACAA	ATTTATGAGGGGGTGGTGCG	109
**MDHAR**	XM044548068.1	TCGGAGCTGGAGGAATTTGG	TGATCTCATCACCCCCACCA	102
**NOX**	XM044485283.1	CCATGCCAAGAATGGTGTCG	ATACGGGTGCTTTGAGGCAA	109
**PCS1**	AF093752.1	TACTGCATCTAAGGCGGCAG	GTGAGCAGATTGTCGCAGC	106
**Actin**	GQ339780.1	CCAGTACTGCTGACTGAGGC	ACACGATACCTGTTGTGCGT	151
**25Sr RNA**	KY129794.1	ACAATTGGTCATCGCGCTTG	GCGTTCAGTCATAATCCGGC	72
**AP5**	XM037577304.1	AATTGTTTCAAGTGGCCGGG	TGGCCGACCGAAATGATGAA	116
**UPF3**	Ta044476849.1	CTGATAGCTCAACCCCGCAG	GGCCTCATTTCGCTGGTCTT	86
**Eef-1α**	Ta044534770.1	GCGTGACATGAGACAAACGG	AAACCTTCTGCTAGATAGATGCCT	125

Supplementary Table S3. Absolute biometric values of length (cm•plant^{-1}) and weight (g•plant^{-1}) of shoots and roots of Razek and Chili durum wheat cultivars exposed to Cd (0 (control), 5 and 50 µM) for 72 h. Values represent the mean ± S.D. of five biological replicates and different letters indicate significant differences between treatments and between the cultivars (italic) (p<0.05; one-way ANOVA).

		SHOOT		ROOT	
	Cd (µM)	Length (cm•plant^{-1})	Weight (g•plant^{-1})	Length (cm•plant^{-1})	Weight (g•plant^{-1})
RAZEK	0	22.3 ± 2.6$^{a,\mathit{a}}$	0.36 ± 0.02$^{b,\mathit{a}}$	11.7 ± 1.2$^{a,\mathit{a}}$	0.19 ± 0.01$^{a,\mathit{a}}$
	5	19.1 ± 1.8$^{ab,\mathit{a}}$	0.32 ± 0.06$^{b,\mathit{a}}$	11.2 ± 1.6$^{a,\mathit{a}}$	0.17 ± 0.03$^{ab,\mathit{a}}$
	50	16.7 ± 1.2$^{b,\mathit{a}}$	0.27 ± 0.07^{c}	9.3 ± 1.0$^{a,\mathit{a}}$	0.14 ± 0.03$^{b,\mathit{a}}$
CHILI	0	30.8 ± 3.7$^{a,\mathit{b}}$	0.55 ± 0.07$^{a,\mathit{b}}$	14.0 ± 1.4$^{a,\mathit{a}}$	0.29 ± 0.03$^{a,\mathit{b}}$
	5	26.5 ± 2.1$^{b,\mathit{b}}$	0.34 ± 0.09$^{b,\mathit{a}}$	11.7 ± 2.3$^{a,\mathit{a}}$	0.18 ± 0.05$^{b,\mathit{a}}$
	50	23.1 ± 2.4$^{b,\mathit{b}}$	0.29 ± 0.08$^{b,\mathit{a}}$	11.0 ± 1.6$^{a,\mathit{a}}$	0.15 ± 0.04$^{b,\mathit{a}}$

Supplementary Table S4. Biothiol concentration (nmol/g FW) cysteine (Cys), glutathione and phytochelatin 3 (PC3) concentration (nmol/g FW) in root, and GSH and phytochelatins (PCs) in shoot of Chili durum wheat variety exposed to Cd (50 µM) for 0, 24, 48 and 96 h. Values represent the mean ± S.D. of five biological replicates, and green or red boxes indicate significant up- or down- variation relative to 0 h exposure. Different letters mark significant differences between treatments (p<0.05; one-way ANOVA).

(h)	0	24	48	96
**ROOT**				
Cys	n.d. ^{a}	57.2 ± 16.6 ^{b}	72.5 ± 18.3 ^{bc}	76.1± 5.5 ^{c}
GSH	75.8± 26.1 ^{b}	56.2 ± 5.3 ^{ab}	48.8 ± 12.4 ^{a}	42.1± 7.6 ^{a}
PC2	n.d. ^{a}	43.8 ± 10.1 ^{b}	56.6 ± 42.7 ^{b}	95.3 ± 20.6 ^{c}
PC3	n.d. ^{a}	56.8 ± 16.3 ^{b}	81.9 ± 43.7 ^{bc}	114.6 ± 21.6 ^{c}
**SHOOT**				
GSH	286.8 ± 11.3 ^{a}	239.9 ± 62.5 ^{a}	286.7 ± 74.1 ^{a}	268.2 ± 74.6 ^{a}
PCs	n.d. ^{a}	n.d. ^{a}	n.d. ^{a}	n.q. ^{a}

n.d.: not detected.
n.q.: not quantifiable.

Supplementary Table S5. Expression levels of antioxidant and stress responding genes in leaves of plants grown in 0 (control), 5 and 50 μM Cd. Values represent the mean ± S.D. of five biological replicates relative to the control (set at 1.00). Green or red colors indicate respectively significant up- or down-regulation relative to the control ($p < 0.05$).

Razek			GENE	Chili		
Cd treatment (μM)				Cd treatment (μM)		
Control	5	50		Control	5	50
1.00 ± 0.20	8.82 ± 1.76	7.15 ± 1.41	*pAPX*	1.00 ± 0.15	5.64 ± 1.15	1.13 ± 0.15
1.00 ± 0.07	3.23 ± 0.21	2.49 ± 0.40	*GSH*	1.00 ± 0.13	2.81 ± 0.28	0.58 ± 0.05
1.00 ± 0.13	5.43 ± 0.88	4.80 ± 0.82	*HSP70*	1.00 ± 0.04	3.33 ± 0.39	0.60 ± 0.05
1.00 ± 0.19	6.29 ± 0.94	3.37 ± 0.76	*MnSOD*	1.00 ± 0.24	4.99 ± 1.09	0.90 ± 0.19
1.00 ± 0.13	4.19 ± 0.91	1.77 ± 0.24	*CAT*	1.00 ± 0.08	3.27 ± 0.49	1.04 ± 0.08
1.00 ± 0.13	4.71 ± 0.71	4.12 ± 0.37	*ABA*	1.00 ± 0.06	3.34 ± 0.66	2.63 ± 0.19
1.00 ± 0.11	5.09 ± 0.41	5.04 ± 0.64	*ATG8*	1.00 ± 0.11	3.79 ± 0.38	0.76 ± 0.18
1.00 ± 0.12	1.94 ± 0.18	0.82 ± 0.17	*GR*	1.00 ± 0.11	1.54 ± 0.12	0.91 ± 0.16
1.00 ± 0.10	5.68 ± 0.70	5.77 ± 0.76	*GPX*	1.00 ± 0.03	2.83 ± 0.43	0.57 ± 0.06
1.00 ± 0.16	4.62 ± 1.07	2.59 ± 0.26	*HSP17.6*	1.00 ± 0.17	1.11 ± 0.14	0.29 ± 0.03
1.00 ± 0.18	3.18 ± 1.26	5.24 ± 1.17	*DHAR1*	1.00 ± 0.37	2.43 ± 0.84	3.52 ± 0.41
1.00 ± 0.27	3.48 ± 1.04	3.49 ± 0.64	*MDHAR*	1.00 ± 0.36	1.17 ± 0.19	1.70 ± 0.34
1.00 ± 0.31	4.52 ± 1.31	3.26 ± 0.94	*NOX*	1.00 ± 0.16	1.60 ± 0.43	1.45 ± 0.23
1.00 ± 0.14	2.46 ± 0.36	1.75 ± 0.42	*PCS1*	1.00 ± 0.06	1.66 ± 0.40	0.44 ± 0.05

Supplementary Table S6. Expression levels of antioxidative and stress responding genes in the leaves of Chili plants exposed to 50 μM Cd for 0, 24, 48, and 96 h. Values represent the mean ± S.D. of four biological replicates relative to 0 h (set at 1.00). Green or red colors indicate respectively significant up- or down-regulation relative to the control ($p < 0.05$).

Time of exposure				GENE
**0 h**	**24 h**	**48 h**	**96 h**	
1.00 ± 0.09	0.76 ± 0.02	0.94 ± 0.16	1.21 ± 0.01	***pAPX***
1.00 ± 0.15	1.72 ± 0.32	1.34 ± 0.35	1.07 ± 0.79	***GSH***
1.00 ± 0.07	1.06 ± 0.40	1.27 ± 0.65	1.27 ± 0.59	***HSP70***
1.00 ± 0.10	0.75 ± 0.26	2.62 ± 1.18	2.85 ± 0.74	***MnSOD***
1.00 ± 0.11	0.78 ± 0.14	0.64 ± 0.18	0.80 ± 0.09	***CAT***
1.00 ± 0.11	3.14 ± 0.47	3.43 ± 1.25	1.74 ± 1.04	***ABA***
1.00 ± 0.16	2.81 ± 0.41	2.75 ± 0.71	1.92 ± 1.45	***ATG8***
1.00 ± 0.11	1.66 ± 0.24	1.20 ± 0.23	0.88 ± 0.58	***GR***
1.00 ± 0.11	2.68 ± 0.30	2.56 ± 0.78	1.66 ± 1.30	***GPX***
1.00 ± 0.28	2.45 ± 0.31	1.61 ± 0.66	0.51 ± 0.10	***HSP17.6***
1.00 ± 0.20	3.76 ± 0.56	4.87 ± 1.26	3.08 ± 2.16	***DHAR1***
1.00 ± 0.16	2.26 ± 0.42	1.67 ± 0.32	1.52 ± 0.89	***MDHAR***
1.00 ± 0.37	2.00 ± 0.41	2.24 ± 0.81	1.33 ± 0.86	***NOX***
1.00 ± 0.24	1.94 ± 0.19	1.45 ± 0.18	0.47 ± 0.16	***PCS1***

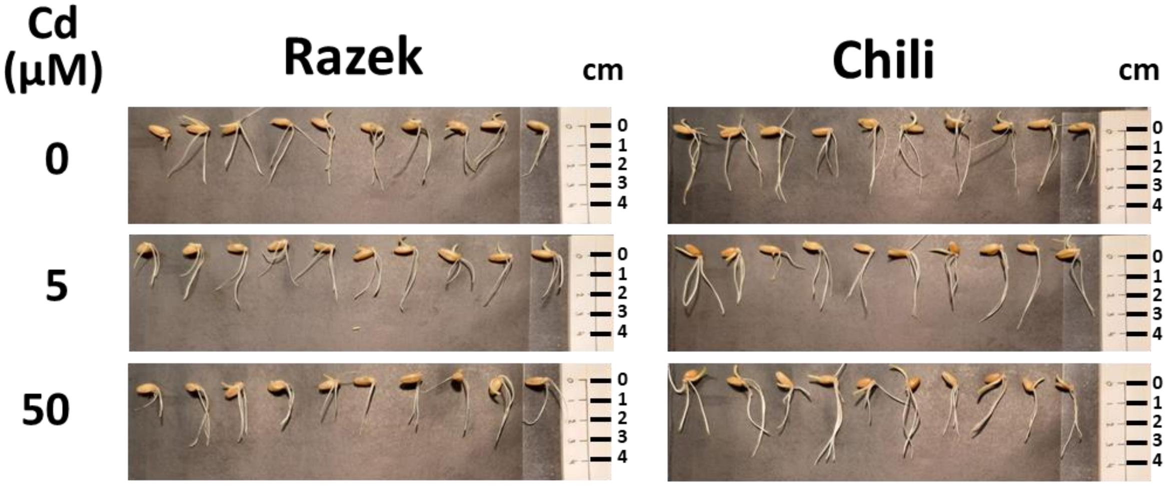

Supplementary Figure S1. Seed germination test of durum wheat cultivars Razek and Chili in control (0), 5 and 50 µM Cd solutions, as example of the preliminary tests used for screening of Cd-tolerant durum cultivars.

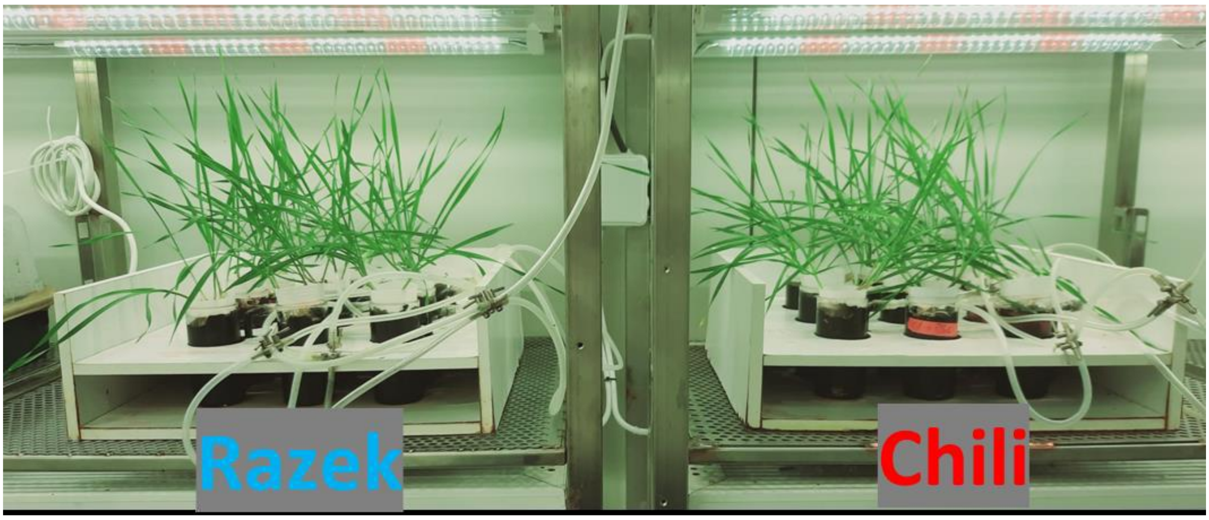

Supplementary Figure S2. Hydroponic system setup using graduated cylinders filled with Hoagland's nutrient solution (150 mL) for the growth of durum wheat cultivars Razek and Chili.

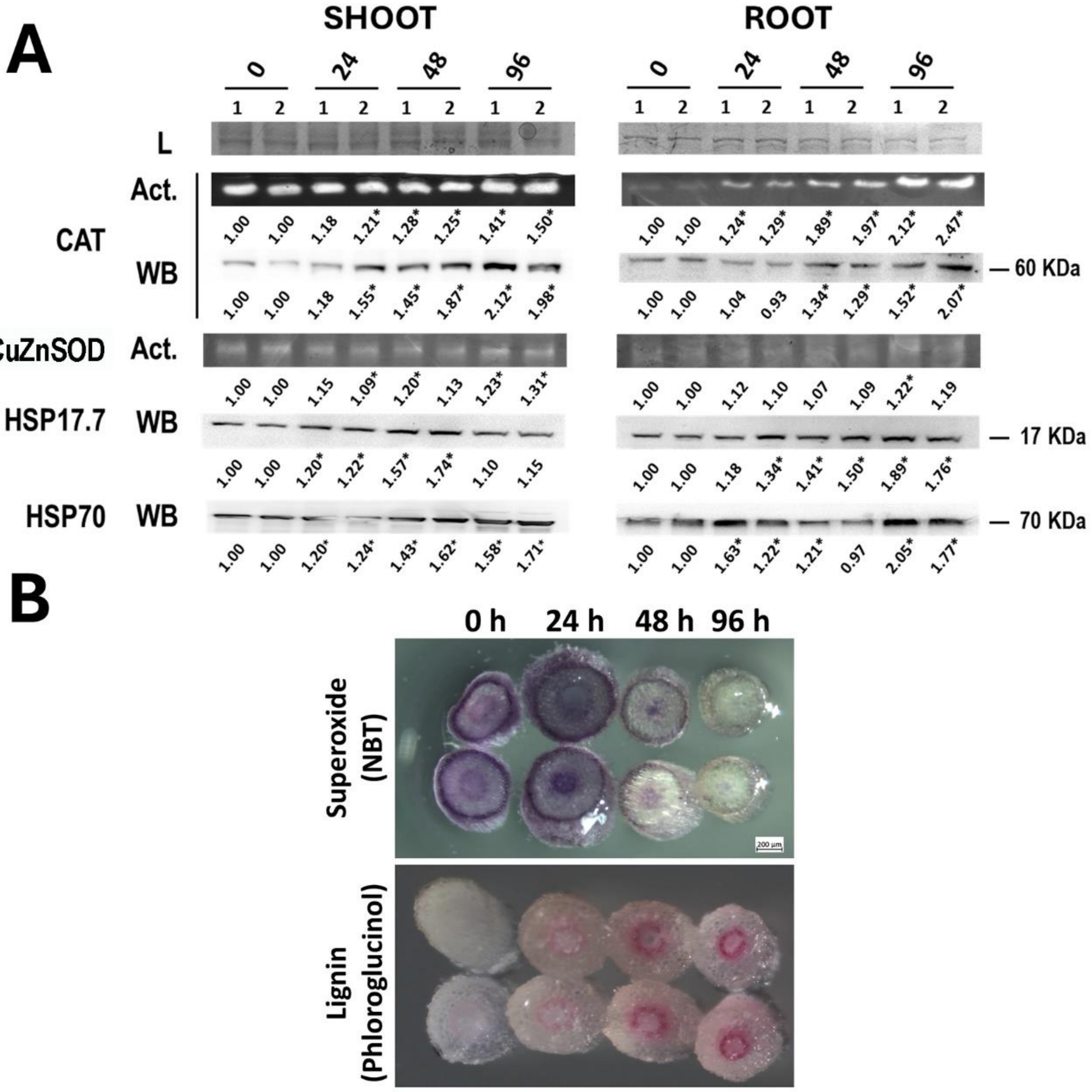

Supplementary Figure S3. A) Time course Cd-treatment experiment: Analysis of catalase (CAT), Cu/Zn superoxide dismutase (SOD), small heat shock protein 17.7 (HSP17.7) and heat shock protein 70 (HSP70), enzymatic activities (Act.) and/or immunodetection (WB) in shoots and roots of Chili durum wheat exposed to 50 μM Cd for 0, 24, 48 and 96 h. Two biological independent replicates were analysed, and figures represent normalised signal (with protein loading (L) after Coomassie blue staining) relative to control samples. Asterisks highlight ± 20% variation with the control. **B)** Colorimetric staining of superoxide (NBT) and lignin (phoroglucinol) in Chili durum wheat plants exposed to 50 μM Cd for 0, 24, 48 and 96 h.
